# Silent mutations at codons 65 and 66 in reverse transcriptase alleviate indel formation and restore fitness in subtype B HIV-1 containing D67N and K70R drug resistance mutations

**DOI:** 10.1093/nar/gkv128

**Published:** 2015-03-12

**Authors:** Sushama Telwatte, Anna C. Hearps, Adam Johnson, Catherine F. Latham, Katie Moore, Paul Agius, Mary Tachedjian, Secondo Sonza, Nicolas Sluis-Cremer, P. Richard Harrigan, Gilda Tachedjian

**Affiliations:** 1 Centre for Biomedical Research, Burnet Institute, Melbourne, Victoria 3004, Australia; 2 Department of Microbiology, Monash University, Clayton, Victoria 3800, Australia; 3 Department of Infectious Diseases, Monash University, Melbourne, Victoria 3004, Australia; 4 Centre for Population Health, Burnet Institute, Melbourne, Victoria 3004, Australia; 5 CSIRO Biosecurity Flagship, Australian Animal Health Laboratory, Geelong, Victoria 3220, Australia; 6 Department of Microbiology and Immunology, University of Melbourne, Parkville, Victoria 3010, Australia; 7 Department of Medicine, University of Pittsburgh School of Medicine, Pittsburgh, PA 15261, USA; 8 British Columbia Centre for Excellence in HIV/AIDS, Vancouver, BC V6Z1Y6, Canada

## Abstract

Resistance to combined antiretroviral therapy (cART) in HIV-1-infected individuals is typically due to nonsynonymous mutations that change the protein sequence; however, the selection of synonymous or ‘silent’ mutations in the HIV-1 genome with cART has been reported. These silent K65K and K66K mutations in the HIV-1 reverse transcriptase (RT) occur in over 35% of drug-experienced individuals and are highly associated with the thymidine analog mutations D67N and K70R, which confer decreased susceptibility to most nucleoside and nucleotide RT inhibitors. However, the basis for selection of these silent mutations under selective drug pressure is unknown. Using Illumina next-generation sequencing, we demonstrate that the D67N/K70R substitutions in HIV-1 RT increase indel frequency by 100-fold at RT codons 65–67, consequently impairing viral fitness. Introduction of either K65K or K66K into HIV-1 containing D67N/K70R reversed the error-prone DNA synthesis at codons 65–67 in RT and improved viral replication fitness, but did not impact RT inhibitor drug susceptibility. These data provide new mechanistic insights into the role of silent mutations selected during antiretroviral therapy and have broader implications for the relevance of silent mutations in the evolution and fitness of RNA viruses.

## INTRODUCTION

Nucleoside and nucleotide reverse transcriptase (RT) inhibitors (NRTIs) and nonnucleoside reverse transcriptase inhibitors (NNRTIs) are essential components of combined antiretroviral therapy (cART) to control human immunodeficiency virus (HIV) infection (1). NRTIs such as zidovudine (ZDV), stavudine (d4T), lamivudine (3TC), emtricitabine and tenofovir (TFV) are analogs of naturally occurring deoxyribonucleoside triphosphates (dNTPs), which inhibit HIV RT DNA polymerization by acting as chain terminators of nucleic acid synthesis (2). In contrast, NNRTIs such as nevirapine (NVP) are a group of amphiphilic compounds that function as allosteric inhibitors of HIV type 1 (HIV-1) RT DNA polymerization (2).

Despite the efficacy of cART, HIV can rapidly evolve to become drug resistant, a process that is potentiated by suboptimal adherence. In resource-rich settings such as North America and Europe, recent data estimate between 9 and 15% of transmitted drug resistance in virus isolated from HIV-1-infected, antiretroviral-naive individuals (3,4). Furthermore, in low- and middle-income countries where cART is being rapidly scaled up, restricted drug options and access to cART, inconsistencies in drug supply and suboptimal levels of viral load testing for monitoring (5) contribute to the emergence and transmission of drug-resistant HIV-1, which represents a major limiting factor in the efficacy of cART (6). Despite advances in the development of HIV-1 inhibitors, the majority of individuals in low- and middle-income countries are still receiving first-line regimens containing thymidine analogs ZDV and d4T (7) and as such, the emergence of thymidine analog mutations (TAMs) threatens the efficacy of cART in these populations (7).

The emergence of HIV with reduced drug susceptibility is typically due to the selection of nonsynonymous mutations in the nucleotide sequence that result in amino acid changes in viral proteins targeted by drugs. Treatment with ZDV and d4T leads to the emergence of TAMs at RT codons 41, 67, 70, 210, 215 and 219 (8–11). Importantly, the accumulation of TAMs confers cross-resistance to most NRTIs (12). While HIV drug resistance mutations confer a replication advantage in the presence of drug, they typically decrease viral fitness in the absence of inhibitor (13–15). Consequently, additional nonsynonymous compensatory mutations are often selected that potentiate drug resistance and/or increase viral fitness, e.g. L210W (10,11) and K219Q/E (9) that potentiate ZDV resistance in the context of other TAMs (16–18).

In addition to nonsynonymous or ‘amino acid changing’ TAMs, we have previously shown that synonymous RT mutations, namely K65K and K66K, in HIV-1 subtype B are more prevalent in cART-experienced compared to naive individuals and are strongly co-selected with TAMs (19). While these silent mutations, comprising a codon change from AAA to AAG, are selected in subtype B strains during cART (19), they exist as a natural polymorphism in HIV-1 subtype C isolates (20). These polymorphisms are reported to be associated with a more rapid selection of the K65R TFV-resistance mutation in HIV-1 subtype C compared to subtype B (20). This increased selection of K65R is mediated by a template-dependent dislocation mechanism during plus-strand DNA synthesis occurring on a homopolymeric run of six A-nucleotides at RT codons 63–65 (21). In contrast, the corresponding homopolymeric stretch of A's in HIV-1 subtype B spans codons 65–66 of RT. Similar to subtype B, an identical mononucleotide run features in HIV-1 subtypes A, D, G, CRF_AG and CRF_AE, which together with subtype B, constitute a significant proportion of the HIV-1 burden worldwide (22). Emergence of drug-resistant viruses containing the TAMs D67N/K70R in these subtypes creates a run of eight A nucleotides in the RNA template between nucleotides 2742 and 2749 (relative to HXB2) of RT. The presence of the K65K or K66K silent mutations disrupts this homopolymeric A region, and we have demonstrated that these mutations alleviate recombinant HIV-1 RT pausing during cDNA synthesis of this region *in vitro* (19), although the impact of our biochemical findings on HIV-1 replication is unknown.

The emergence of the K65K/K66K silent mutations during drug therapy (19) supports the notion that these mutations confer a replicative advantage. Possible advantages to viruses harboring K65K or K66K are that they increase fitness of HIV-1 subtype B strains and/or decrease their susceptibility to antiviral drugs. In this study, we investigated whether K65K and K66K affect viral fitness as well as susceptibility to RT inhibitors in the context of TAMs. We show that these silent mutations confer a fitness advantage, even in the absence of drug selection pressure and that they do not affect drug susceptibility. Consistent with the ability of silent mutations to reverse RT pausing observed in biochemical assays due to the TAMs D67N/K70R (19), we demonstrate that K65K and K66K decrease the frequency of deleterious insertion and deletion mutations (i.e. indels) introduced in the HIV-1 genome during virus replication that consequently could alleviate the fitness defect due to TAMs. This is the first study to report that silent mutations, selected *in vivo*, mediate their effects on the viral RNA primary sequence to directly impact RT function and virus replication.

## MATERIALS AND METHODS

### Cell culture

MT-2 cells obtained through the NIH AIDS Research Reagent Program (NIH ARRRP), Division of AIDS, NIAID, NIH from Douglas Richman, were maintained in RF-10 medium comprising Roswell Park Memorial Institute (RPMI) 1640 (Invitrogen Life Technologies, Carlsbad, CA, USA), 10% (v/v) heat-inactivated fetal bovine serum (Sigma-Aldrich, Castle Hill, NSW, Australia), 100 U/ml penicillin, 100 μg/ml streptomycin and 2 mM L-glutamine. TZM-bl cell line was obtained from the NIH ARRRP contributed by Dr John C. Kappes, Dr Xiaoyun Wu and Tranzyme Inc. The 293 cell line was obtained from the NIH ARRRP contributed by Dr Andrew Rice. The 293T cell line (CRL-3216) was obtained from American Type Culture Collection (ATCC, Manassas, VA, USA). TZM-bl, 293 and 293T cells were maintained in DMEM-10 medium comprising Dulbecco modified Eagle medium (DMEM) supplemented as above. Human peripheral blood mononuclear cells (PBMC) were isolated from buffy coats (obtained from the Australian Red Cross Blood Bank, Melbourne, Australia) using Ficoll-Paque (Amersham Biosciences, Piscataway, NJ, USA) as previously described (23). Isolated PBMC were stimulated in RF-10 medium supplemented with 10 U/ml recombinant interleukin-2 (Roche, Basel, Switzerland) and 10 μg/ml of 0.2 μm filter-sterilized phytohaemaglutinin-P (PHA-P; Remel Inc., Lenexa, KS, USA) in a dry-heat sterilized silicon-coated polytetrafluoroethylene (Teflon) pot (Savillex, Minnetonka, MN, USA) to elicit cell activation and proliferation over 2–4 days.

### Drugs

ZDV, TFV, abacavir (ABC), NVP and saquinavir (SQV) were obtained through the NIH ARRRP and prepared as 10 mM stocks in dimethyl sulfoxide except for TFV, which was solubilized in dH_2_O. Stocks were stored at −20°C until use.

### Plasmids and virus production

The pDRNLXN construct, derived from pDRNL that contains the subtype B NL4.3 infectious molecular clone of HIV-1 (24), was engineered with silent mutations that introduce *Xba*I and *Not*I restriction sites at nucleotides 2319 and 3938, respectively (25). HIV-1 molecular clones containing mutations in the RT coding region of pDRNLXN were generated by site-directed mutagenesis using the QuikChange Multi Site-Directed Mutagenesis Kit (Stratagene, La Jolla, CA, USA) according to the manufacturer's instructions. Mutagenic oligonucleotide ‘RH1’ (5′-AAAGAAAAAAAACAGTACTAGATGGAGAAAATTAGTAGATTTCAG-3′) was used to introduce the TAMs D67N (GAC- AAC) and K70R (AAA-AGA) in the RT coding region to generate the construct pHIV_TAM_. pHIV_TAM_ was used as the template to generate pHIV_TAMK65K_ and pHIV_TAMK66K_ containing D67N/K70R and the additional silent mutation at codon 65 (AAA to AAG) and codon 66 (AAA to AAG), using the oligonucleotides ‘RH3’ (5′-CCAGTATTTGCCATAAAGAAGAAAAACAGTACTAGATGGAG-3′) and ‘RH2’ (5′-AAGAAAAAGAACAGTACTAGATGGAGAAAATTAGTAGATTTCAG-3′), respectively. All HIV-1 constructs were verified by nucleotide sequencing.

### Generation of HIV-1 by transfection of 293T cells

Twenty-four hours prior to transfection, 3.5 × 10^6^ 293T cells in 10 ml of DMEM-10, were seeded in 75 cm^2^ tissue culture flasks. Ten micrograms of pDRNLXN (hereinafter referred to as pHIV_WT_), pHIV_TAM_, pHIV_TAMK65K_ and pHIV_TAMK66K_ were introduced into 293T cells by the calcium phosphate co-precipitation method as published (26) with the following modifications: supernatants were clarified by low-speed centrifugation at 700 x *g* for 30 min, followed by filtration through a 0.2-μm Minisart filter (Sartorius, Goettingen, Germany) and stored at −80°C until use.

### Infectivity assays

The titer of HIV-1 stocks generated by transfection was determined by end-point dilution in MT-2 cells as previously described (26). Cells were scored as either positive or negative for virus-specific cytopathic effects (CPE) at day 6 postinfection. The 50% tissue culture infectious dose (TCID_50_) was calculated using the Kärber formula (27). Virus infectivity was also determined by quantifying the number of blue forming TZM-bl cells using a β-galactosidase assay as published (26). Virus production was quantified using the RETRO-TEK HIV-1 p24 Antigen ELISA kit (Zeptometrix Corporation, Buffalo, NY, USA) according to manufacturer's instructions.

### Phenotypic drug susceptibility assays

Virus at 100 TCID_50_ was used to infect MT-2 cells (6000 cells/well) in the presence of noncytotoxic concentrations of ZDV, TFV, ABC and NVP. Drug susceptibility of HIV_WT_ and mutant viruses (HIV_TAM_, HIV_TAMK65K_ and HIV_TAMK66K_) was determined by measuring cell viability as the readout for virus replication as previously described (23). Percentage inhibition of virus replication was calculated at each drug concentration as published (28). Drug susceptibility assays were also performed in TZM-bl cells using luciferase activity as the readout for virus replication as published (23). The 50% effective concentration (EC_50_) was determined by the sigmoidal dose response, variable slope nonlinear regression analysis of the log_10_ concentration of inhibitor versus the percentage inhibition of virus replication using GraphPad Prism (Version 6.0a) as published (23).

### Growth competition assays in MT-2 cells and PBMC

Growth competition of HIV-1 strains was performed in MT-2 cells and in PBMC isolated from multiple donors with the same pool of cells used for each set of assays using methods based on a previous study (29). To initiate the competition assay, MT-2 cells at 2 × 10^6^ cells/ml were infected with 1000 TCID_50_ of each competing virus for 2 h at 37°C in 5% CO_2_. Following infection, the volume was increased to 10 ml with RF-10 without drug or containing 2 μM ZDV or 5 μM TFV. Cells were monitored by microscopy over 3–6 days and once virus-specific CPE affected 75% of the cells, supernatants were clarified and cells harvested for subsequent purification of genomic DNA and sequencing of total viral DNA. Progeny viruses in culture supernatants were serially passaged by inoculating 2 × 10^6^ uninfected MT-2 cells with 0.9 ml supernatant from the previous passage in the absence or presence of drug. Initiation of growth competition assays in PBMC was achieved by infecting cells with a 0.2 multiplicity of infection (MOI) of HIV_TAM_ and either HIV_TAMK65K_ or HIV_TAMK66K_ in the absence or presence of drug and cultured for 7 days at 37°C in 5% CO_2_. An equal volume of cell suspension was harvested for genomic DNA extraction and sequencing, and the culture supernatant was used for the next passage. Uninfected PHA-stimulated PBMC (2.5 × 10^6^) from the same HIV seronegative donor and 5 ml of IL-2 media (RF-10 supplemented with 10 U/ml recombinant IL-2) were added to propagate the culture weekly.

The RT region of the initial virus inoculum was sequenced to confirm the proportions of competing viral strains. The QIAamp Viral RNA Mini Kit (Qiagen, Hilden, Germany) was used to purify HIV-1 genomic RNA from the inoculum after treatment with 10 U/ml of DNase I and 40 U/ml of Benzonase for 1 h at 37°C to remove plasmid DNA carryover. Reverse transcriptase-polymerase chain reaction (RT-PCR) was performed with the primers 5′V3-NL (5′-GTAGGACAGTATGATCAGATA-3′) and 3′V2-NL (5′-TTGTAGGGAATGCCAAATTCC-3′) using the Mastercycler Nexus Gradient Thermal Cycler instrument (Eppendorf, Hamburg, Germany) as follows: 30 min RT reaction at 50°C, 2 min Superscript III Platinum Taq enzyme activation at 94°C, followed by 35 cycles at 94°C for 15 s, 52°C for 30 s and 68°C for 2 min, and a final extension at 68°C for 5 min. DNA sequencing was performed on viral DNA present in extracted total cellular DNA from each passage to determine the proportions of each virus. Total DNA was extracted using the QIAamp DNA Blood Mini Kit (Qiagen) and a 2.2 kb region spanning the HIV-1 RT coding region was amplified by PCR with HotStarTaq Plus (Qiagen) and primers 5′V3-NL and 3′V2-NL using the following cycle conditions: 95°C for 5 min, followed by 40 cycles at 95°C for 30 s, 50°C for 30 s and 72°C for 2 min and a final extension of 72°C for 7 min. PCR products were purified using the High Pure PCR Product Purification Kit (Roche, Basel, Switzerland) and sequenced using the primers Sp1aV2 (5′-CTGAAAATCCATACAATACTC-3′), RSp2a (5′-CACATCCAGTACTGTTAC-3′), Rev3041 (5′-CTAAAAGGCTCTAAGATTTTTGTC-3′) and ST3246-NL (5′-GAACTCCATCCTGATAAATG-3′). Levels of competing virus were determined as described previously (29,30) by calculating the peak height of nucleotides on electropherograms at RT codons 65 or 66 as a proportion of the sum of the peak heights using the formula *P_n_ = M_n_ / (W_n_ + M_n_)* where *P*_n_ represents the relative proportion of the mutant at day *n, W* represents the peak height for the WT nucleotide and *M* is the mutant peak height at the corresponding nucleotide position. ‘*P_n_*’ values were utilized to generate fitness vectors, whereby the ratio *P_n_/P_0_*, was plotted as a function of time expressed as days in culture, where *P*_0_ is the relative proportion of the mutant at day 0. Linear regression analyses were performed to determine the line of best fit for *n* ≥ 3 independent assays for each condition and to assess whether the slopes were significantly different from the neutral case (i.e. *m* = 0). The Runs test was performed for each independent assay to confirm that the data did not significantly deviate from linearity. A 2.1 kb fragment spanning *gag* from nucleotides 496 to 2589 (relative to HXB2 coordinates) was amplified using the cycling conditions for HIV-1 RT PCR amplification with primers, M667 (5′-GGCTAACTAGGGAACCCACTG-3′) and 3′RSpPR, (5′-GCTTTAATTTTACTGGTACAG-3′). The amplimer was sequenced using the primer, Int-2A5 (5′-GTAATACCCATGTTTTCAGCATTA-3′) to confirm the absence of extraneous mutations selected in *gag* during virus passage.

### Analysis of intracellular RT efficiency by quantitative PCR

MT-2 cells (2 × 10^5^) were infected with 0.1 MOI of HIV_WT_, HIV_TAM_, HIV_TAMK65K_ or HIV_TAMK66K_. Viral stocks were pre-treated with DNase I and Benzonase as above to remove plasmid DNA carryover from transfections. An additional viral sample was heat-inactivated at 65°C for 45 min and served as a negative control to indicate DNA carryover. Treated viral stocks were added to cells in a 48-well plate in a final volume of 200 μl of RF-10 for 2 h and then media volume was increased to 700 μl for the remainder of the infection. Cells were harvested at the appropriate time points by centrifugation at 1000 x *g* for 5 min and washed with phosphate buffered saline without magnesium and calcium [PBS(-)]. Total DNA was extracted from cell pellets using the QIAamp DNA Blood mini kit (Qiagen) and DNA eluted in 100 μl Buffer AE. DNA was treated with a final concentration of 0.1 units/μl of *Dpn*I at 37°C for 2 h prior to analysis of RT intermediates by quantitative PCR (qPCR).

The production of early and late reverse transcription (RTn) intermediates was determined by qPCR using primer pairs targeting the RU5 (hRU5-F2: 5′-GCC TCA ATA AAG CTT GCC TTG A-3′ and hRU5-R: 5′-TGA CTA AAA GGG TCT GAG GGA TCT-3′) and U5ψ (MH531: 5′-TGT GTG CCC GTC TGT TGT GT-3′ and MH532: 5′-GAG TCC TGC GTC GAG AGA TC-3′) regions, respectively (31). qPCR amplifications were performed using 1 x Brilliant II SYBR qPCR Master Mix (Agilent Technologies, Santa Clara, CA), 300 nM of each primer, 30 nM ROX reference dye and 20 ng of DNA in a final reaction volume of 25 μl. Samples were amplified using a MX3000 cycler at 95°C for 10 min, 40 cycles at 95°C for 30 s and 60°C for 1 min followed by melt-curve analysis. Standard curves were created from pHIV_WT_ for early and late HIV-1 RTn products, which were serially diluted in carrier genomic DNA derived from MT-2 cells at a final concentration of 10 ng/μl.

### Single- and multiple-cycle infections

MT-2 cells (1 × 10^6^) were infected with HIV_WT_, HIV_TAM_, HIV_TAMK65K_ or HIV_TAMK66K_ (pre-treated to remove contaminating DNA as described above) at an MOI of 1 for 4–6 h at 37°C. Following infection, the virus inoculum was removed by centrifuging cells at 1000 x *g* for 5 min, followed by washing cells with PBS(-), and replenishing with RF-10. At 30 h postinfection, culture supernatant was harvested for subsequent p24, infectivity and RT activity analysis. The cell pellet was washed with PBS(-) and stored at −80°C. Cells infected with heat-inactivated virus or infected in the presence of 2 μM SQV were included as controls. For multiple-cycle infections, 2 × 10^6^ MT-2 cells were infected with 1000 TCID_50_ units of HIV_WT_, HIV_TAM_, HIV_TAMK65K_ and HIV_TAMK66K_. For propagation over 19 days, 900 μl of virus supernatant was transferred from the previous passage to uninfected MT-2 cells every 3–4 days.

### DNA preparation for next-generation amplicon sequencing

Total DNA was purified from HIV-1-infected MT-2 cells and *Dpn*I treated as described above using the QIAamp DNA Blood mini kit (Qiagen). A 156 bp fragment encompassing the silent mutations in the RT coding region (2659 to 2815 of HXB2) was amplified using the primers DSFwd (5′-TCG TCG GCA GCG TCA GAT GTG TAT AAG AGA CAG ATTTGT ACA GAA ATG GAA AAG GAA G-3′) and DSRev (5′-GTC TCG TGG GCT CGG AGA TGT GTA TAA GAG ACA GCCCAG AAA TCT TGA GTT CTC-3′), which incorporated nucleotide sequences (Nextera adapters, underlined) required for subsequent deep sequencing analysis. PCR amplifications were performed under conditions specifically designed to limit PCR errors and recombination (32). Reactions contained 200 μM dNTPs, 1 μM each primer, 0.8 units of Phusion Hotstart II DNA polymerase (Thermo Fisher Scientific, Waltham, MA), 10 μl of extracted genomic DNA and 1 x Phusion HF buffer in a final volume of 20 μl. Cycle conditions were 98°C for 30 s, 25 cycles at 98°C for 10 s, 65°C for 20 s and 72°C for 30 s, followed by a final extension at 72°C for 10 min. To minimize PCR founder effects and costs, each sample from the single-cycle infections was amplified in triplicate and a total of nine reactions from triplicate amplifications from three independent experiments were pooled for deep sequencing analysis as previously published (33,34). The target sequence was amplified as above from total DNA in triplicate and pooled. To determine the background nucleotide substitutions and ‘indels’ (insertions and deletions) introduced by the PCR and deep sequencing conditions, the 156 bp RT region from plasmids pHIV_WT_, pHIV_TAM_, pHIV_TAMK65K_ and pHIV_TAMK66K_ (150 ng) was amplified in triplicate with the DSFwd and DSRev primers under identical PCR conditions used for the total DNA template and pooled. qPCR analysis was used to confirm that this input amount of plasmid DNA was within the log-linear phase of amplification after 25 PCR cycles, ensuring that template saturation did not occur. Library preparation was performed by addition of unique Illumina Nextera XT adapters to each pooled PCR sample using Q5 High Fidelity DNA polymerase (New England Biolabs, Ipswich, MA, USA). DNA sequencing was performed using 150 bp paired-end reads on an Illumina MiSeq instrument at Micromon Next-Generation Sequencing Facility (Monash University, Melbourne, Australia).

### Next-generation sequencing analysis

Verification of read quality, read trimming and mutation frequency was performed using CLC Genomics Workbench version 6.5.1 (CLC bio, Aarhus, Denmark). First, sequence data quality for each paired-end dataset was assessed using the Create Sequencing QC Report tool. Next, the CLC Genomics Workbench Trim Sequences algorithm was used to remove the Illumina Nextera adapter sequences followed by the RT primer sequences. The Merged Overlapping Pairs algorithm was used to merge the forward and corresponding reverse sequence reads to produce a 100% overlap, resulting in improved confidence of the read quality. Median PHRED quality score in the region of interest (i.e. 108 bp sequence representing HXB2 nucleotides 2685–2792) was greater than 60, where there is a 1 in a million probability that a base call is incorrect. Unmerged sequences were discarded. The merged paired-end reads were then trimmed based on read quality using the Trim Sequences algorithm using the default settings except that Quality Limit was set at 0.001, ‘yes’ was selected for Quality Trim and the reads were not trimmed on ambiguous nucleotides. Selected reads matching the region of interest were sorted so that all reads were a uniform length of 108 bp starting from the same position in the RT sequence. Finally, the quality of the trimmed reads was assessed using the Create Sequencing QC Report algorithm. The number of high-quality merged paired-end reads remaining ranged from 215 139 to 1.25 × 10^6^ after sequence trimming.

To analyze mutations introduced during intracellular RTn and their frequencies, the reads were mapped to their corresponding reference sequence (e.g. reads from infections with HIV_WT_ were mapped to the WT RT sequence) using the Map Reads to Reference algorithm in CLC Genomics Workbench. The default settings were used except that nonspecific handling was set to ‘ignore’. The Quality-Based Variant Detection algorithm was then used to detect insertions, deletions, single nucleotide variants (SNV) and multiple nucleotide variants (MNV) using the default CLC Genomics Workbench settings except that minimum variant frequency was set to 0.005%, variant filter was deselected and ploidy was set at 1. The overall mutation frequency within the 108 bp RT region for HIV_WT_ and mutant strains subjected to single-cycle and long-term culture were calculated by dividing the sum of the frequencies of indels, SNV and MNVs by the length of the sequenced amplimer (Supplementary Table S2).

### Statistical analyses

The Wilcoxon rank-sum test was used to determine whether there were significant differences between RT inhibitor EC_50_ values for HIV-1 mutants in drug susceptibility assays and differences in viral protein production, infectivity and RTn intermediates. Binomial *z*-tests were performed to determine the statistical difference in the mutation frequency at specific nucleotide positions in the viral genome. To account for the multiple post-hoc comparisons and implied increase in Type-I error, Bonferroni correction was applied to inferential tests and statistical significance assessed at *P* < 0.005. Where joint effects were sought for a mutation, weighted (by reads) generalized linear modeling (assuming a binomial distribution and log link function) was performed and postestimation Wald tests used to determine both joint effects and the differences between mutation types. All statistical analyses were performed using GraphPad Prism version 6.0a (GraphPad Software Inc.) and Stata version 13 (StataCorp L.P.).

## RESULTS

### RT silent mutations K65K or K66K do not potentiate resistance to RT inhibitors in the context of TAMs D67N/K70R

TAMs including D67N and K70R are significantly associated with K65K or K66K, suggesting that these silent mutations contribute to strategies employed by the virus to escape from the inhibitory effects of antiretroviral drugs *in vivo* (19). Accordingly, we investigated whether HIV-1 harboring K65K or K66K (AAA to AAG change) in the presence of the TAMs, D67N and K70R (HIV_TAMK65K_ and HIV_TAMK66K_, respectively), potentiated resistance to RT inhibitors.

The susceptibility of HIV_TAMK65K_ and HIV_TAMK66K_ was evaluated in the TZM-bl reporter cell line against the antiretroviral drugs, TFV and ZDV that target RT, in parallel with WT (HIV_WT_) and mutant virus carrying the TAMs D67N/K70R alone (HIV_TAM_; Table 1). We found that HIV_TAM_ was 2.6- and 1.9-fold less susceptible to ZDV (*P* = 0.004, *n* = 5) and TFV (*P* = 0.03, *n* = 6), respectively, compared to HIV_WT_, consistent with previously published data (35). However, we found no significant differences in the TFV or ZDV susceptibility of HIV_TAM_ and either HIV_TAMK65K_ or HIV_TAMK66K_ (*P* > 0.05, *n* ≥ 3 for both) (Table 1). To increase our ability to detect small changes in drug susceptibility under conditions of multiple rounds of virus infection, we performed assays in MT-2 cells. In addition to testing susceptibility to TFV and ZDV, we expanded the drugs to include ABC and NVP. The TFV and ZDV susceptibility data were similar to that obtained in TZM-bl cells, while none of the mutants demonstrated decreased susceptibility to ABC and NVP compared with HIV_WT_ (Table 1). Taken together, these data demonstrate that neither the K65K nor the K66K silent mutation potentiates drug resistance conferred by TAMs.

**Table 1.** K65K and K66K do not potentiate resistance to RT inhibitors

	ZDV				TFV				ABC		NVP	
	TZM-bl		MT-2		TZM-bl		MT-2		MT-2		MT-2	
	Mean EC_50_ ± SE (μM)^a^	Fold change^b^	Mean EC_50_ ± SE (μM)^a^	Fold change^b^	Mean EC_50_ ± SE (μM)^a^	Fold change^b^	Mean EC_50_ ± SE (μM)^a^	Fold change^b^	Mean EC_50_ ± SE (μM)^a^	Fold change^b^	Mean EC_50_ ± SE (μM)^a^	Fold change^b^
HIV_WT_	0.36 ± 0.06 (5)	1.0	0.08 ± 0.02 (3)	1.0	2.50 ± 0.50 (6)	1.0	1.46 ± 0.11 (5)	1.0	0.53 ± 0.10 (6)	1.0	0.08 ± 0.02 (3)	1.0
HIV_TAM_	0.86 ± 0.17 (5)	2.6^c^	0.53 ± 0.16 (3)	5.8^c^	4.60 ± 1.03 (6)	1.9^c^	2.46 ± 0.26 (5)	1.9^c^	0.53 ± 0.10 (6)	1.1	0.07 ± 0.01 (3)	0.9
HIV_TAMK66K_	0.92 ± 0.25 (5)	2.5^c^	0.53 ± 0.17 (3)	5.9^c^	4.85 ± 0.58 (6)	2.6^c^	2.45 ± 0.26 (5)	1.8^c^	0.62 ± 0.13 (6)	1.1	0.08 ± 0.01 (3)	1.2
HIV_TAMK65K_	0.72 ± 0.10 (5)	2.1^c^	0.7 ± 0.23 (3)	7.8^c^	4.09 ± 0.47 (6)	2.2^c^	2.15 ± 0.40 (5)	1.5	0.45 ± 0.12 (6)	0.8	0.04 ± 0.01 (3)	0.6

^a^Mean 50% effective concentration ± standard error (EC_50_ ± SE) calculated from at least three independent experiments. The number of independent experiments is indicated in parenthesis.

^b^Fold change of EC_50_ value relative to HIV_WT_.

^c^EC_50_ value is significantly different from WT (*P*<*0.05*) as determined using the Wilcoxon rank-sum test.

### RT silent mutations K65K or K66K alleviate a fitness defect conferred by the TAMs D67N/K70R

Based on our previous data showing that K65K and K66K alleviate pausing of recombinant HIV-1 RT during cDNA synthesis of a synthetic RNA template containing D67N/K70R (19), we hypothesized that these silent mutations overcome a HIV-1 fitness defect conferred by the TAMs D67N/K70R. Accordingly, we performed growth competition assays in MT-2 and PBMC with HIV_TAM_ and the HIV_TAMK65K_ or HIV_TAMK66K_ mutants in both the presence and absence of TFV or ZDV.

**Figure 1.**
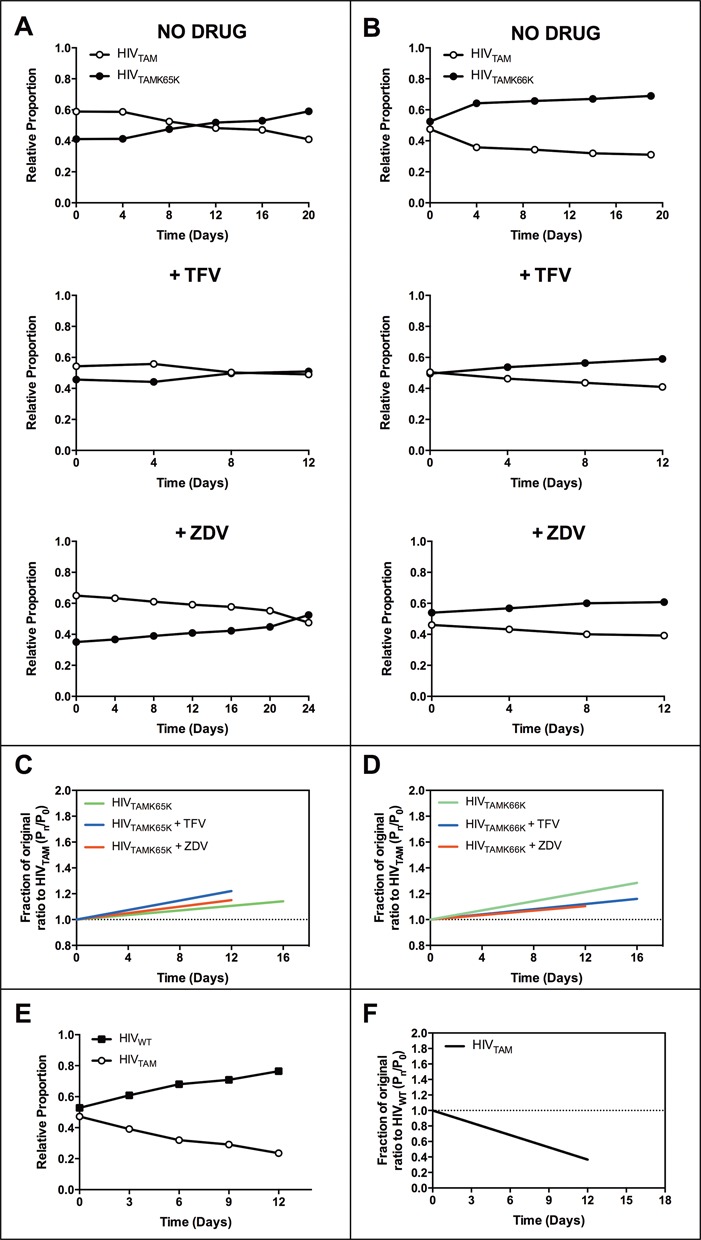
Growth competition dynamics between HIV_TAM_ and HIV_TAMK65K_ or HIV_TAMK66K_ and between HIV_WT_ and HIV_TAM_ in MT-2 cells. Representative plots from *n* = 1 growth competition assays performed in MT-2 cells with (**A**) HIV_TAM_ and HIV_TAMK65K_ or (**B**) HIV_TAM_ and HIV_TAMK66K_, passaged in the absence or presence of either 5 μM TFV or 2 μM ZDV as indicated. Average fitness vectors representing the rate of change in the proportion of (**C**) HIV_TAMK65K_ and (**D**) HIV_TAMK66K_ relative to HIV_TAM_ compared to their ratio in the initial inoculum (day 0) plotted relative to the days in culture. The relative fitness measure, which represents the vector slope, or linear coefficient (*m*), was determined by linear regression by plotting the ratios of mutant virus over time relative to their starting proportion in culture using data from *n* ≥ 3 independent growth competition assays performed in MT-2 cells. Line of best fit is presented across time points for which there were *n* ≥ 3 data points for each assay condition. The Runs test was performed on data from independent assays and revealed no significant deviations from linearity. A positive or negative linear coefficient indicates a fitness gain or loss, respectively. (**E**) Representative plot from growth competition assays with HIV_WT_ and HIV_TAM_. Average fitness vector representing the rate of change in the proportion of (**F**) HIV_TAM_ relative to HIV_WT_ compared to the ratio in the initial inoculum (day 0) and plotted relative to the days in culture.

HIV_TAMK65K_ (Figure 1A) and HIV_TAMK66K_ (Figure 1B) were more fit relative to HIV_TAM_ in both the absence and presence of TFV and ZDV in assays performed in MT-2 cells. To generate fitness vectors, the proportion of HIV_TAMK65K_ or HIV_TAMK66K_ relative to HIV_TAM_ at each passage was expressed relative to their respective proportions at day 0 for each independent assay (36). Linear regression analyses were performed to determine the line of best fit for data derived from *n* ≥ 3 independent growth competition assays for HIV_TAMK65K_ and HIV_TAMK66K_ (i.e. fitness vectors) and to assess whether the slopes were significantly different from the neutral case (i.e. *m* = 0). Analysis using the Runs test confirmed no significant deviations from linearity in the datasets analyzed. Positive slopes were observed for all HIV_TAMK65K_ and HIV_TAMK66K_ fitness vectors in assays performed in MT-2 cells (Figure 1C and D, respectively), indicating that these viral strains are more fit relative to HIV_TAM_. Linear regression analysis revealed all vector slopes (*m*) were significantly different (*P* ≤ 0.001) from the neutral case where *m =* 0 (Table 2). Although consistently observed in replicate *in vitro* assays, the effect of the silent mutations was subtle, and typically required multiple passages to become apparent. This was in contrast to the fitness defect conferred by the HIV_TAM_ mutant, which was outcompeted by HIV_WT_ much more rapidly (Figure 1E) and resulted in a steeper, negative slope for the HIV_TAM_ fitness vector relative to HIV_WT_ (*m = -*0.051 ± 0.004, *n* = 3; Figure 1F and Table 2).

**Table 2.** Relative fitness of TAM mutants

Variant	Cells	Mutations	Fitness (vector slope) ± SE^c^		
			No drug	ZDV (1 μM)	TFV (5 μM)
HIV_TAM_^a^	MT-2	D67N/K70R	-0.051 ± 0.004^d^*	-	-
HIV_TAMK65K_^b^	MT-2	D67N/K70R/K65K	0.009 ± 0.001^d^*	0.010 ± 0.001^d^*	0.018 ± 0.002^d^*
HIV_TAMK66K_^b^	MT-2	D67N/K70R/K66K	0.018 ± 0.002^d^*	0.009 ± 0.002^d^*	0.010 ± 0.002^d^*
HIV_TAMK65K_^b^	PBMC	D67N/K70R/K65K	0.008 ± 0.0004^e^*	0.005 ± 0.0007^e^*	0.017 ± 0.002^e^*
HIV_TAMK66K_^b^	PBMC	D67N/K70R/K66K	0.005 ± 0.0003^e^*	0.007 ± 0.0004^e^*	0.006 ± 0.001^e^*

^a^Relative to HIV_WT_.

^b^Relative to HIV_TAM_.

^c^SE, standard error of the slope.

^d^*n* ≥ 3 independent assays.

^e^*n* = 2 independent assays.

*P < 0.001, significance of deviation from *m =* 0 calculated by linear regression analyses using the mean relative ratios from *n* ≥ 2 independent assays.

**Figure 2.**
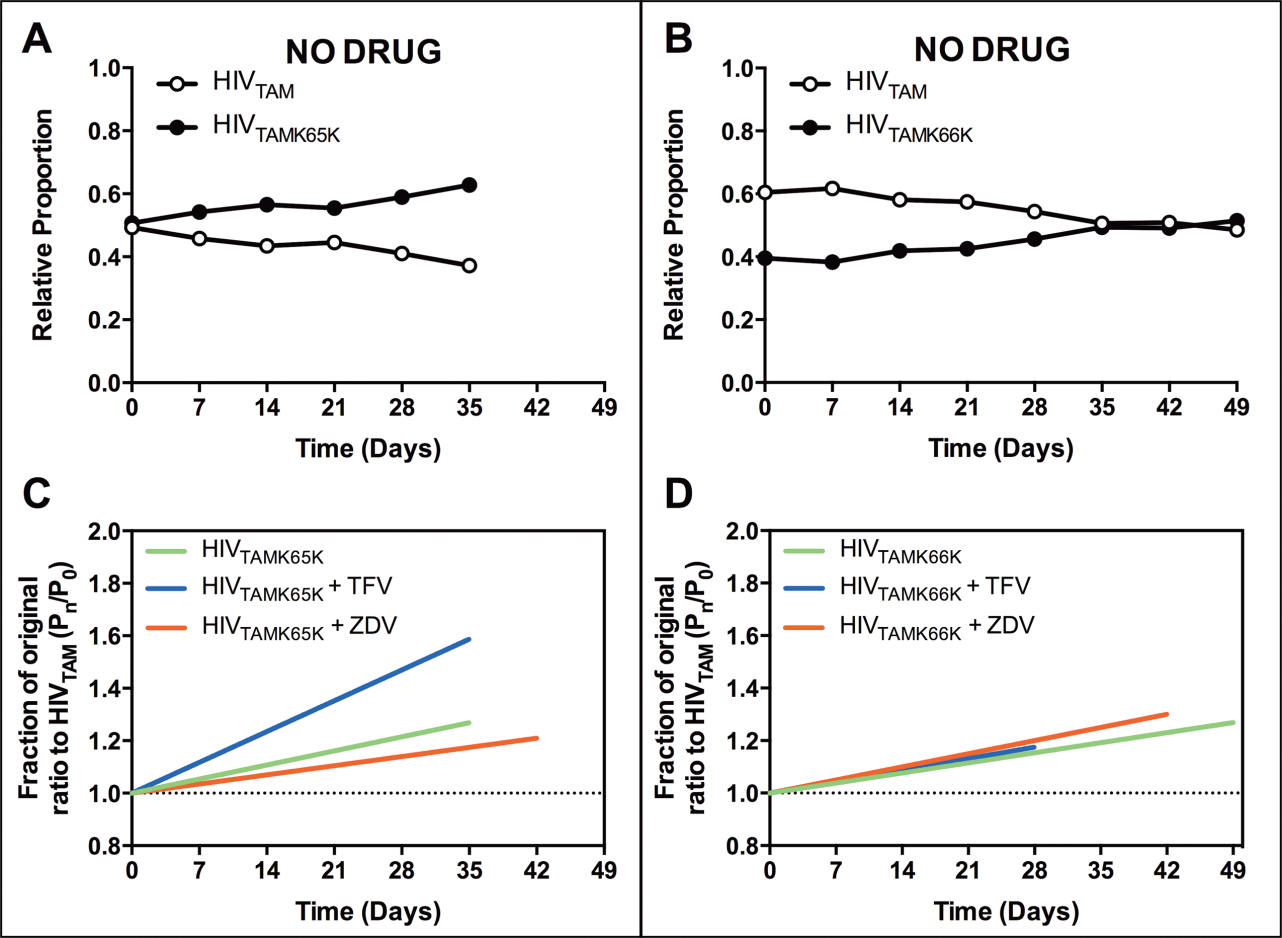
Growth competition dynamics between HIV_TAM_ and HIV_TAMK65K_ or HIV_TAMK66K_ mutants in PBMC. Representative plots from growth competition assays performed in PBMC for (**A**) HIV_TAM_ and HIV_TAMK65K_ and (**B**) HIV_TAM_ and HIV_TAMK66K_ passaged in the absence of drug. Fitness vectors for (**C**) HIV_TAMK65K_ and (**D**) HIV_TAMK66K_ mutants were calculated from growth competition assays performed in the presence and absence of either 5 μM TFV or 1 μM ZDV. Data were from *n* = 2 independent assays.

A similar fitness trend was observed in growth competition assays performed in PBMC where HIV_TAMK65K_ and HIV_TAMK66K_ outcompeted HIV_TAM_ in the absence (Figure 2A and B) and presence of either ZDV or TFV (Supplementary Figure S1). The fitness vectors for HIV_TAMK65K_ and HIV_TAMK66K_ relative to HIV_TAM_ in growth competition assays performed in PBMC in the absence and presence of RT inhibitors were all positive (Figure 2C and D) and the vector slopes were significantly different (*P* ≤ 0.001) from 0 (Table 2), supporting our initial observation in MT-2 cells.

Sequencing of the entire RT coding region in samples from the final passage of each growth competition assay in MT-2 cells and PBMC confirmed both the retention of the silent mutations and TAMs and the absence of any additional mutations that may have arisen during multiple rounds of infection, particularly in the presence of drug. In addition, part of the capsid coding region was sequenced, which confirmed the absence of the H87Q mutation reported to confer a replication advantage to HIV-1 in cyclophilin A-rich cells such as MT-2 (37). Taken together, these data demonstrate that the emergence of the K65K or K66K silent mutations in the context of TAMs D67N/K70R in subtype B HIV-1 alleviates the fitness defect caused by these TAMs in the absence or presence of AZT and TFV.

### TAMs D67N/K70R in the absence or presence of silent mutations decrease the production of early and late RTn products

HIV-1 harboring TAMs D67N/K70R in the absence or presence of the K65K or K66K silent mutations generated by transfection did not lead to significant decreases in viral infectivity, steady-state virion-associated RT protein levels or RT activity relative to WT virus (Supplementary Figures S2 and S3). In contrast, viruses containing TAMs did show reduced infectivity and virion-associated RT activity relative to WT virus in a single-cycle infection of MT-2 cells (Supplementary Figure S4) consistent with our fitness data in Figure 1E and F. However, there was no significant difference among the HIV_TAM_ mutants in their virion-associated RT activity relative to p24 due to the presence of the silent mutations (Supplementary Figure S4B). This might be explained by the smaller effect of the silent mutation containing variants, HIV_TAMK65K_ and HIV_TAMK66K_ relative to HIV_TAM_ observed by fitness vectors (Figure 1C and D) compared to the fitness vector for HIV_TAM_ relative to HIV_WT_ (Figure 1F).

**Figure 3.**
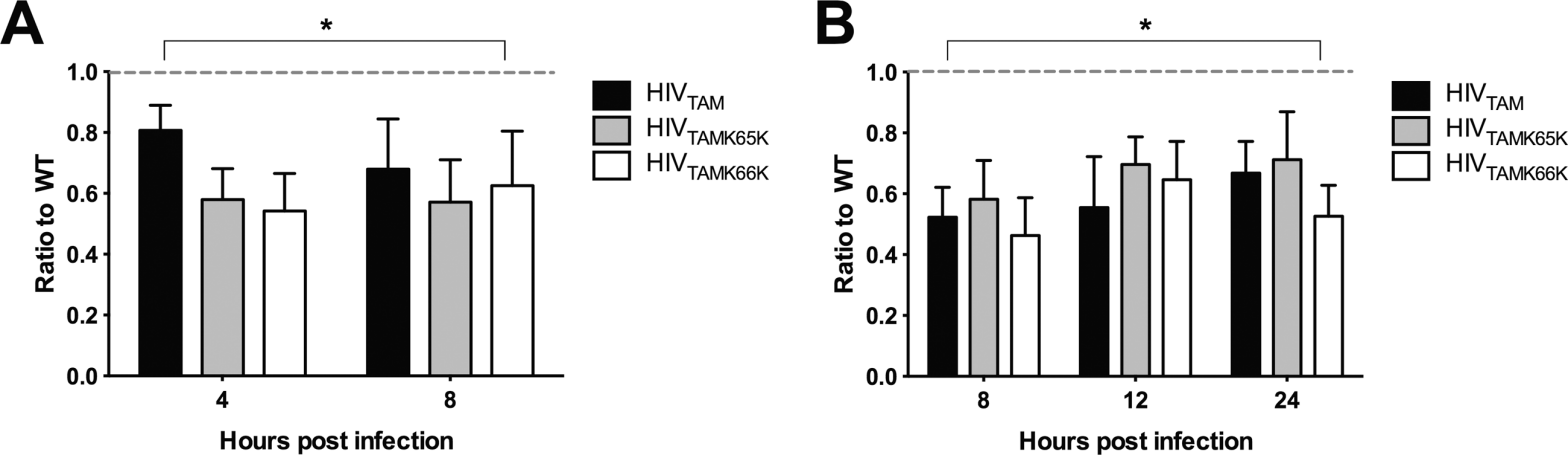
Production efficiency of early and late RTn intermediates. Expression of (**A**) early RTn products and (**B**) late RTn products, relative to HIV_WT_ (WT) as determined by qPCR. The RTn intermediates produced from purified total DNA extracted from cells were assessed at 4, 8, 12 and 24 h postinfection from *n* ≥ 3 independent assays. Error bars represent SEM. The expression of early and late RTn intermediates for all mutants was significantly different from HIV_WT_ (*P <* 0.05). No significant differences were observed between HIV_TAM_ and HIV_TAMK65K_ or HIV_TAMK65K_ (*P* > 0.05). Statistical analysis was performed using the Wilcoxon rank-sum test.

We next assessed whether the D67N/K70R TAMs confer an intracellular RTn defect, and whether this defect is reversed by K65K or K66K. Early (minus strand strong stop) and late RTn intermediates were detected by qPCR at various times after a single-cycle infection of MT-2 cells (Figure 3). We observed a significant decrease in the production of early (Figure 3A) and late (Figure 3B) RTn products for HIV_TAM_ compared to HIV_WT_ (*P =* 0.04 and *P =* 0.006, respectively, *n* ≥ 3). However, no significant differences in the production of early or late RTn products were detected between HIV_TAM_ and either HIV_TAMK65K_ or HIV_TAMK66K_ (*P* = 0.33 and *P* = 0.20, respectively, *n* ≥ 3). Furthermore, the ratio of early to late RTn products did not reveal alterations to the overall efficiency of production of RTn intermediates between these viruses (data not shown). Heat-inactivated controls were routinely negligible for detection of HIV-1 DNA by qPCR. These data show that the TAMs D67N/K70R decrease RTn efficiency. However, our data indicate that either the fitness advantage conferred by silent mutations does not affect RTn efficiency or that the single-cycle infection assay we employed lacked the sensitivity to measure subtle effects conferred by K65K and K66K.

### K65K and K66K rescue increased frequency of indels caused by TAMs during single- and multiple-cycle infections

**Figure 4.**
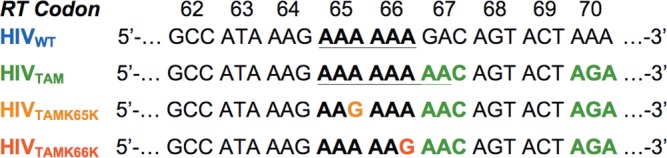
Nucleotide sequence of RT codons 62–70 for WT and mutant HIV-1. Nucleotide sequence showing D67N and K70R and the single nucleotide changes resulting in K65K and K66K. The homopolymeric stretch of six adenines in HIV_TAM_ and eight adenines in HIV_TAM_ are shown in boldface and underlined. The introduction of a silent mutation at either codon 65 (HIV_TAMK65K_) or 66 (HIV_TAMK66K_) disrupts this homopolymeric stretch of nucleotides. The D67N and K70R mutations are shown in green.

Introduction of the TAM D67N in the HIV-1 RT of clade B isolates extends a homopolymeric run of A nucleotides from six to eight (Figure 4). We have previously reported that this substantial homopolymeric stretch causes the clade B HIV-1 RT to pause during DNA synthesis on RNA templates harboring D67N/K70R in cell-free assays (19). Moreover, introducing either K65K or K66K in this sequence disrupts the homopolymeric stretch (Figure 4), which alleviates RT pausing *in vitro* (19). Given that we did not observe differences in RTn efficiency due to silent mutations in a single-cycle infection (Figure 3) and that homopolymeric nucleotide regions are associated with increased rates of synthesis errors (38,39), we next considered the possibility that the change in nucleotide sequence impacts on the fidelity of RTn.

Accordingly, we examined whether the TAMs D67N/K70R increase errors introduced by HIV-1 RT during intracellular RTn that are alleviated by silent mutations K65K and K66K. To investigate this, we performed both single- and multiple-cycle (over 19 days) infections of WT and TAM mutant viruses in MT-2 cells and sequenced total HIV-1 DNA from infected cells using the Illumina next-generation sequencing platform to determine the presence of mutations in a 108 base pair (bp) amplimer containing the RT region of interest. Plasmid proviral clones pHIV_WT_, pHIV_TAM_, pHIV_TAMK65K_ and pHIV_TAMK66K_ were subjected to the same PCR amplification and deep sequencing protocols as DNA from virus-infected cells to control for mutations introduced during the amplification/sequencing steps. The input plasmid concentration was confirmed to be below that required for template saturation because DNA was extracted at a cycle number within the log-linear phase of amplification (data not shown). The raw reads for each condition were evaluated to select those of the highest quality, utilizing a strict cut-off to control for base calling accuracy, trimmed to remove the Nextera adapters and locus-specific primer sequences, followed by merging forward and the corresponding reverse sequence reads to further enhance read quality. Our analysis included a minimum of 215 139 overlapping merged reads, with a median of 931 644 reads per template. The overall mutation frequency, including insertions, deletions, SNV and MNV within the 108 bp RT region for HIV-1 strains was determined (Supplementary Table S1). The average mutation frequency for HIV_WT_ in a single-cycle infection was 0.059% (i.e. 0.00059 mutations/bp). Accordingly, mutations were considered above background if their frequency was >0.06%. This stringent threshold is ∼40 times greater than the error rate for HIV-1 RT determined using a HIV-1 vector containing the LacZ α reporter gene (40).

**Figure 5.**
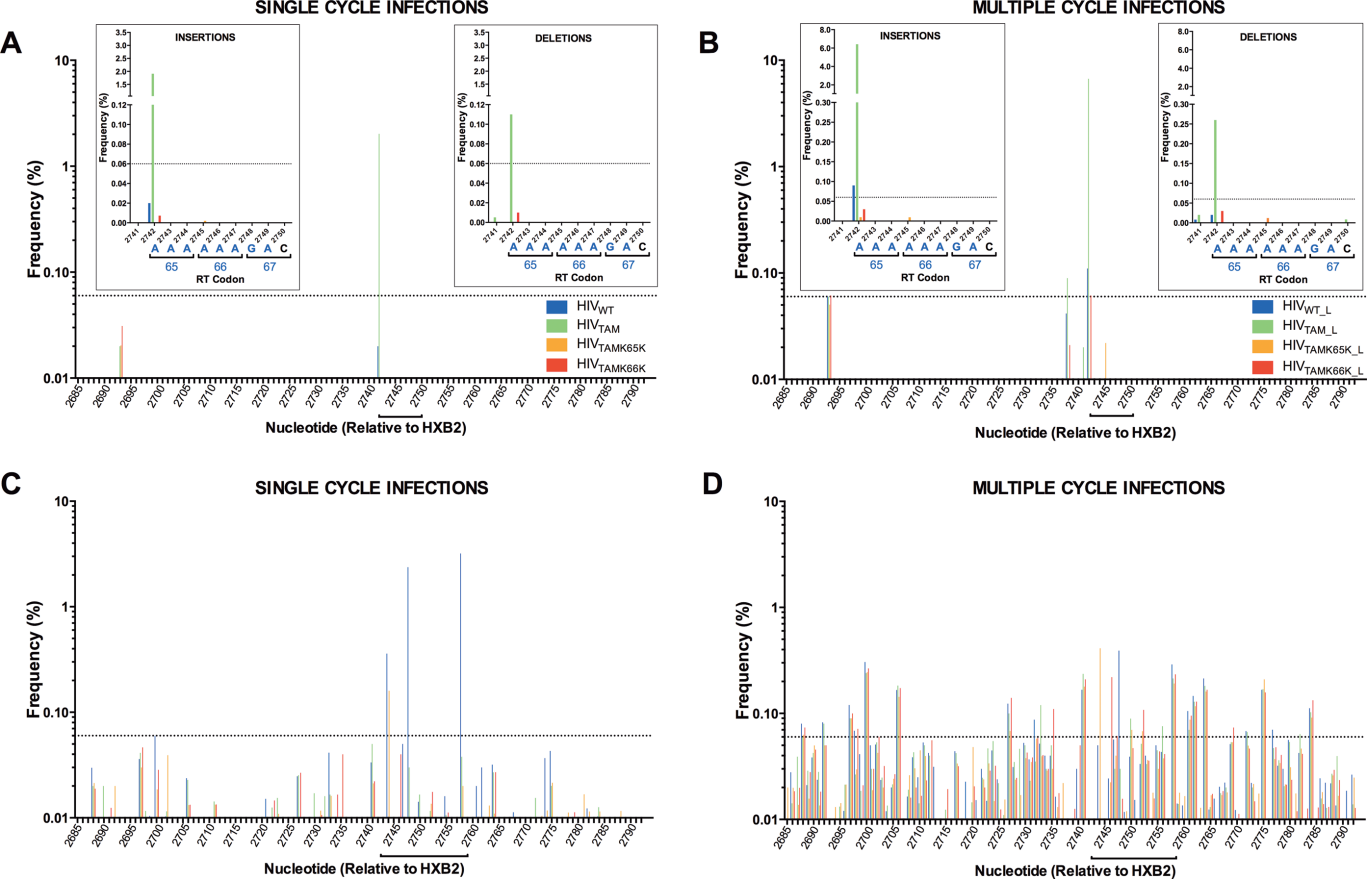
Frequency of indels and single/multiple nucleotide changes in RT codons 46–81 observed in HIV-1 mutants. Next-generation amplicon sequencing data were generated by amplifying a 156 bp region of HIV-1 RT using total DNA purified from MT-2 cells subjected to a single-cycle or multiple-cycle infection with HIV_WT_, HIV_TAM_, HIV_TAMK65K_ or HIV_TAMK66K_. The frequency of reads (defined as the percentage of validated reads that has a mutation compared to the reference sequence) containing indel mutations spanning RT codons 46–81 (HXB2 coordinates 2685–2792) during (**A**) single-cycle infections and (**B**) multiple-cycle infections are shown. The sum of all insertions (single, double, triple and quadruple insertions) or deletions at a particular nucleotide is shown. Inset graphs show the frequencies of insertions and deletions arising during (A) single-cycle and (B) multiple-cycle infections located at nucleotides 2741–2750 corresponding to RT codons 65–67 (underlined) with the HIV_WT_ sequence shown below the x-axis. The sum of all insertions (single, double and triple insertions) or deletions at each nucleotide is shown. The frequency of reads containing either single and/or multiple nucleotide variants spanning RT codons 46–81 (HXB2 coordinates 2685–2792) during (**C**) single-cycle infections and (**D**) multiple-cycle infections. The sum of the frequency of all mutations at each nucleotide for each virus is shown. The mutation frequency was determined by mapping all reads to the corresponding reference sequence and subtracting the mutation frequency detected in the cognate plasmid control at each nucleotide. Frequencies >0.06% were designated as above background (dotted line).

Analysis of HIV_WT_ and mutant strains subjected to single- and multiple-cycle infections revealed that most of the indel mutations that were present above background levels were located in our region of interest between nucleotides 2742 and 2750 corresponding to RT codons 65–67 (Figure 5A and B, respectively, underlined). Likewise, the highest frequencies of SNV/MNV for single- and multiple-cycle infections were detected within the region spanning RT codons 65–70 (nucleotides 2742–2759; Figure 5C and D, respectively, underlined). Unsurprisingly, the differences in SNV and MNV frequencies within RT codons 65–70 compared to the flanking regions observed for multiple-cycle infections were less dramatic than those for single-cycle infections likely due to mutations not being advantageous in the absence of drug (and thus potentially reverting to WT) and the increased frequency of other nondeleterious mutations that accumulate in multiple-cycle infections (Figure 5C and D). Also, the pattern of appearance of indels for all viruses at nucleotide 2742, corresponding to the first nucleotide of the homopolymeric stretch of nucleotides commencing at codon 65, remained comparable between the two infection conditions (Figure 5A and B).

Infection with HIV_TAM_ resulted in a 101-fold (single-cycle, *P* < 0.0001) and 61-fold (multiple-cycle, *P <* 0.0001) increase in indels at nucleotide 2742 compared to HIV_WT_, which was completely reversed by the presence of silent mutations at either K65K or K66K (Figure 5A and B, inset). In a single-cycle infection assay, HIV_TAM_ showed a greater frequency of A insertions (1.91%; comprising 1.86% single A and 0.05% double AA insertions) within the eight-base homopolymeric run at RT codons 65–67 (nucleotides 2742–2750) compared to HIV_WT_ (0.02%, *P* < 0.0001), HIV_TAMK65K_ (no detected insertions, *P* < 0.0001) and HIV_TAMK66K_ (0.007%, *P* < 0.0001) (Figure 5A and Supplementary Table S2). This represents a 96-fold increase in single and double A insertions for HIV_TAM_ compared to HIV_WT_. For the long-term 19-day infection, a 71-fold increase in the frequency of A insertions was observed at the corresponding nucleotides for HIV_TAM_L_ as compared to HIV_WT_L_ (6.42 versus 0.09%, *P <* 0.0001) (Figure 5B inset and Supplementary Table S3). These included single, double, triple and quadruple A insertions observed at frequencies of 5.77, 0.55, 0.09 and 0.01%, respectively. Similar to the single-cycle infections, insertions detected for HIV_TAMK65K_L_ (0.00985%) and HIV_TAMK66K_L_ (0.03%) were comparable to HIV_WT_L_ (0.09%) and were significantly lower (*P <* 0.0001 for both) than the frequency observed for HIV_TAM_L_ (6.42%).

In addition to insertions, we also observed an increase in the frequency of single A nucleotide deletions in HIV_TAM_ compared to HIV_WT_ at nucleotide 2742 for both single- (Figure 5A inset; 0.11% versus no detected insertions, *P* < 0.0001) (Supplementary Table S2) and multiple-cycle infections (Figure 5B inset; 0.26 versus 0.02%, *P* < 0.0001) (Supplementary Table S3). In sharp contrast to HIV_TAM_, the frequency of deletions in HIV_TAMK65K_ and HIV_TAMK66K_ for both single- and multiple-cycle infections observed in the homopolymeric run of A nucleotides at codons 65–67 were similar to the deletion frequency observed for HIV_WT_ (Figure 5A and B). Taken together, these data demonstrate that the TAMs D67N/K70R significantly increase the frequency of indel mutations at the homopolymeric region starting at nucleotide 2742, by up to 101-fold compared to WT, which is completely reversed by the presence of the silent mutations K65K or K66K.

### Increase in indels observed at other homopolymeric regions in RT

In HIV_WT_, a homopolymeric region of six adenines exists at RT codons 65 and 66 that would be disrupted by K65K and K66K (Figure 4). In virus subjected to long-term passage we observed an elevated frequency of indels for HIV_WT_L_ (0.11%) compared to HIV_TAMK65K_L_ (0.01%) and HIV_TAMK66K_L_ (0.06%) at nucleotide 2742 (Figure 5B and Supplementary Table S3). Further supporting our findings that homopolymeric regions promote indel mutations, we observed an elevated frequency of indels (although around the background frequency of 0.06%) for HIV_WT_ at an upstream homopolymeric region of five adenines commencing at nucleotide 2693 during multiple-cycle infections (see Figure 5B). Notably, the indel frequency for all three mutant viruses at the identical region was comparable to HIV_WT_ (Figure 5B: mean frequency of 0.058 ± 0.003% SEM). Although below the defined threshold (0.06%), a similar pattern of increased indel frequency at the corresponding homopolymeric region was observed for the majority of viruses subjected to single-cycle infections (Figure 5A: mean = 0.018 ± 0.006% SEM at nucleotide 2693). The only other increase in indel frequency above background occurred at the third nucleotide of codon 63 (nt 2738) in HIV_TAM_ (0.089%) during the multiple-cycle infection (Figure 5B). In HIV_TAM_ the A nucleotide at position 2738 marks the beginning of a stretch of 11 A's interrupted by a single G nucleotide at position 2741 (codon 64) flanked by three upstream and eight downstream A's (Figure 4). This is in contrast to HIV_WT_, HIV_TAMK65K_ and HIV_TAMK66K_, which have only six, two and five A's immediately downstream of the G at nt 2741 (Figure 4). Similar to our observations in the proximal homopolymeric region spanning codons 65–67, the indel frequency detected for HIV_WT_, HIV_TAMK65K_ and HIV_TAMK66K_ at nt 2738 was below background (0.04, 0.0016 and 0.02%, respectively). Our observation that the indel frequency increases at other, albeit less extensive, homopolymeric A regions in HIV-1 RT suggests that the increase in indels is likely a general phenomenon and not necessarily specific to the homopolymeric run introduced by D67N/K70R TAMs.

### HIV-1 harboring TAMs with and without silent mutations do not demonstrate an increase in SNV or MNV compared to WT virus

In contrast to indels, we did not detect any increases in SNV or MNV above the background mutation frequency following a single-cycle infection of HIV_TAM_ that could potentially be alleviated by silent mutations (Figure 5C and D). Surprisingly, the highest nucleotide substitution frequencies above background were observed in HIV_WT_. We detected the G to A mutation at nucleotide position 2748 at a frequency of 2.4% for single-cycle infections (Figure 5C and Supplementary Table S2) resulting in the D67N substitution. We also observed an A to G change at nucleotide 2758 in HIV_WT_ at a frequency of 3.2% resulting in the emergence of the K70R TAM (Figure 5C and Supplementary Table S2). In each case, the frequency of SNV for HIV_WT_ was significantly higher than that observed in the TAM mutant strains, which already contain mutations at these positions (*P* < 0.0001 for nt 2748 and *P* < 0.0001 for nt 2758 relative to all TAM mutants; Supplementary Table S2). We observed a similar trend of identical SNV occurring at the same nucleotides resulting in the D67N (0.39% at nt 2748, *P* < 0.0001) and K70R (0.29% at nt 2758, *P* < 0.0001) in multiple-cycle infections (Figure 5D and Supplementary Table S3). Additionally, MNVs emerged in HIV_WT_ during single-cycle infections, with the most frequent MNV located at nucleotide 2744 (RT codon 65) culminating in the emergence of K65K and D67N in the same read (0.3%; Figure 5C and Supplementary Table S2). Taken together, these data demonstrate that an increase in indel frequency but not SNV or MNV is associated with decreased fitness conferred by the TAMs D67N/K70R, and that silent mutations K65K or K66K completely reverse this indel formation.

## DISCUSSION

This is the first description of synonymous mutations selected *in vivo* in the coding region of a critical HIV-1 enzyme that promote viral fitness. Our data support a novel mechanism underlying the fitness advantage conferred by these synonymous mutations where their effects are mediated through the primary sequence of the viral genome to influence RT functionality. These mutations can directly impact RT activity by increasing the fidelity of DNA synthesis from the viral nucleic acid template. Specifically, the synonymous mutations, K65K and K66K, in the RT of HIV-1 subtype B alleviate fitness defects conferred by the TAMs D67N/K70R. We show using next-generation deep sequencing that the D67N/K70R TAMs in subtype B HIV-1 conferred an approximately 100-fold increase in the frequency of indel mutations within a homopolymeric region at nucleotides spanning RT codons 65–67 during single-cycle infections and approximately 61-fold increase in indels during multiple-cycle infections relative to HIV_WT_. Strikingly, the introduction of either K65K or K66K into the RNA template containing D67N/K70R reversed the propensity for RT to introduce indel mutations that could otherwise lead to the production of defective provirus. Prior *in vitro* studies conducted by Harrigan *et al*. (19) revealed that RT pauses on the homopolymeric tract of eight adenines introduced by D67N/K70R, and that the silent mutations K65K or K66K alleviate this pause. Initially, we anticipated that this would decrease the overall efficiency of reverse transcription. However, the pausing could also be likely due to misincorporation events that temporarily slow reverse transcription and contribute to the decrease in HIV-1 fitness that was observed in this study.

Our studies demonstrate a definitive role for K65K and K66K in rescuing a viral fitness defect mediated by the TAMs, D67N/K70R while having no effect on potentiating NRTI and NNRTI resistance in the context of TAMs. Our finding that D67N/K70R confer a fitness defect in the absence of drug is consistent with previous studies (41–42,35), although the underlying mechanism for this defect is not well described. Quantitative analysis of competition assays revealed that the fitness gains for HIV_TAMK65K_ and HIV_TAMK66K_ relative to HIV_TAM_ were remarkably similar with and without drug (Table 2). This indicates that silent mutations reverse a defect in HIV-1 replication unrelated to the ability of TAMs to decrease drug susceptibility. This is consistent with our data demonstrating that K65K and K66K in the context of D67N/K70R do not alter the susceptibility of virus to ZDV, TFV, ABC or NVP (Table 1). K65K and K66K are located in the coding region of a vital HIV-1 enzyme and act by altering RTn through effects on the primary viral genome sequence. This contrasts with synonymous mutations previously reported in HIV-1, such as Q41 and L44 in gp41, which act to compensate for fitness defects due to enfuvirtide resistance mutations by stabilizing the RNA secondary structure of the Rev-response element (43). Although the size of the fitness gains mediated by K65K or K66K is relatively small compared to that of drug resistance mutations under selective pressure (e.g. D67N relative to WT in the presence of ZDV (35)), over multiple cycles of replication *in vivo* these small fitness gains are likely to be amplified and have clinical implications. Indeed, our initial observation that K65K and K66K are commonly selected in drug-treated individuals (19) provides compelling support for this assertion.

HIV-1 RNA secondary structure can alter protein translation rate and folding (44). However, our previous RNA secondary structure modeling focusing on short HIV-1 RT templates representing codons 55–75 places K65K and K66K in a single-stranded region (19). Our previous mRNA folding prediction analyses are in agreement with the location of RT codons 65 and 66 in a single-stranded loop region in the structure of the entire HIV-1 genome postulated by Watts *et al*. (44). This is consistent with the observation that highly-structured regions of the HIV-1 genome tend to be G-rich, whereas A-rich nucleotide stretches are more likely to be unpaired (45). Taken together, these *in silico* observations suggest that a mechanism involving changes in RNA secondary structure is unlikely to account for the fitness phenotype. However, while we report that the significantly higher indel frequency (e.g. 101-fold for single-cycle infection) observed for HIV_TAM_ is completely reversed by either the K65K or the K66K silent mutation (Figure 5 and Supplementary Tables S2 and S3), we did not observe restoration of HIV_TAM_ infectivity to WT levels in the presence of either silent mutation in a single-cycle assay (Supplementary Figure S4A). Consequently, in light of these infectivity data and the absence of physical experimental data on RNA secondary structure, we cannot exclude the possibility that additional mechanisms (e.g. RNA secondary structure) could contribute to the fitness defect conferred by TAMs and the compensatory effects of silent mutations.

In addition to altering protein translation rate and folding (44), synonymous mutations are known to affect protein expression and ultimately protein function due to altered mRNA splicing (46), changes in mRNA thermodynamic stability (47) and codon usage biases (48). However, the TAMs D67N/K70R and silent mutations are not located near known splice sites in HIV-1 (49) and are unlikely to generate strong splice sites (unpublished data). While there is a consensus exonic splice enhancer (50) that overlaps RT codons 65–70, we did not find that it was appreciably altered by either of the silent mutations (unpublished data). Relevant to codon usage, the HIV-1 RNA genome has a nucleotide composition that is biased toward more A nucleotides (36%) with only 17% C (51). The selection of silent mutations at RT codons 65 and 66 results in an AAA to AAG change, which in humans, are present at frequencies of 24 and 32%, respectively (52). Thus, AAG represents a preferred lysine codon used in humans that could potentially affect viral protein translation in the context of Gag-Pol. Given the subtle effect of the fitness advantage conferred by the silent mutations K65K and K66K that emerges over multiple rounds of infection, it was not surprising that we were unable to detect any differences between HIV_TAM_ and the silent mutation-carrying variants in infectivity, steady-state levels of virion-associated RT and RT activity in virus produced by transfection (Supplementary Figures S2 and S3). Assessment of HIV-1 infectivity and virion-associated RT activity of these mutants in single-cycle infection assays similarly did not reveal any differences, as expected (Supplementary Figure S4). As such, these data do not appear to support the notion that effects on translation, expression or function of RT or codon usage biases are acting to alleviate the fitness defects caused by D67N/K70R. Our qPCR analysis also failed to dissect differences in the efficiency of RTn intermediate production among TAM mutants with and without silent mutations (Figure 3). Thus, we cannot exclude the possibility that the assays we employed may not have the sensitivity to detect subtle effects of silent mutations on protein expression and virus infectivity that may become apparent during multiple rounds of virus replication if cells are multiply infected, which is likely to occur in MT-2 cells that undergo cell-to-cell fusion (53).

Our study is the first to evaluate RTn errors introduced in the HIV-1 proviral DNA during virus replication mediated by the D67N/K70R TAMs and the *in vivo* selected K65K and K66K silent mutations using Illumina next-generation amplicon sequencing. We observed a dramatic 100-fold increase in the frequency of indels, but not nucleotide substitutions at RT codons 65–67 in HIV_TAM_ compared to HIV_WT_ in a single-cycle infection. The algorithm we used to determine mutation frequency positioned all A-indels at the beginning of the 8 A-homopolymeric run commencing at the first nucleotide of RT codon 65. Hence, where the RT slippage or dislocation occurred and the exact location of the indels within this region is unknown.

Our next-generation amplicon sequencing approach minimized both intra- and interassay variability by addressing several potential confounding factors. First, data for each mutant were derived from three independent infections to minimize infection-related variation. PCR amplifications were performed in triplicate for each independent assay and pooled to minimize PCR-related errors and founder effects. Plasmid DNA of an identical sequence was also analyzed in parallel and background mutations subtracted from each sample to control for experimental errors and mutations introduced by polymerases during the PCR amplification and deep sequencing steps. The Illumina platform has minimal interassay variation and the occurrence of Illumina-specific indel error rates is considerably lower (<0.001 per 100 bases) than other platforms such as 454, making it highly suitable for this type of analysis (54,55). Finally, only the highest quality paired reads were analyzed, where the probability of an incorrect base call was 1 in a million.

Our findings differ from those of a previous study that found that the majority of mutations mediated by ZDV-resistant HIV-1 are either SNV or MNV (56). In contrast to our study, the investigators evaluated the forward mutation rate using a HIV-1 vector containing the LacZα gene as a reporter. Notably synonymous mutations cannot be detected using the LacZα method (57). A report by Mulder *et al*. (58) is consistent with our findings of mutation frequencies in HIV_WT_ of 0.059 and 0.047% during single- and multiple-cycle infections, respectively. The investigators performed a 15-day infection of PBMC that yielded a mutation frequency of 0.083 ± 0.02% in WT virus (HIV_NL4.3_). Their mutation frequency is in a similar range to ours, even though it was determined using orthogonal methods involving molecular cloning and Sanger sequencing.

Distinct to our observation regarding the introduction of indels, we did not observe an increase in the frequency of SNV or MNV in HIV_TAM_ that could potentially be reversed by silent mutations. However, we did detect an increase in the frequency of SNV resulting in the emergence of both D67N (2.37%) and K70R (3.2%) in HIV_WT_ (Figure 5C). This is consistent with D67N and K70R substitutions being among the first to appear *in vivo* (59). A similar trend was detected independently in the multiple-cycle infections (Figure 5D). Furthermore, the increased frequency of MNV resulting in the simultaneous emergence of D67N and K65K but not K70R in HIV_WT_ during single-cycle infections suggests that this observation is unlikely to be a result of contamination with our D67N/K70R TAM containing mutants during the deep sequencing protocol. Notably, the G to A mutation at 2748 (leading to D67N change) in HIV_WT_ during single-cycle infections is 6-fold higher than the frequency observed in the multiple-cycle infection. The differences in mutation frequency are likely due to the reverse A to G change occurring at nucleotide 2748 during multiple cycles of infection, with the mutation frequency after 19 days reflecting the equilibrium of G to A and A to G during culture. Transition mutations (i.e. A↔G) are common in HIV replication (60), but in the absence of drug, there is no selective pressure for the retention of either the G to A mutation that leads to the D67N change or indeed the A to G change that results in K70R at nucleotide 2758 (Figure 5C); consequently, it is unlikely that these mutations would be overrepresented in multiple-cycle infections.

In agreement with our finding that the homopolymeric stretch of eight nucleotides at RT codons 65–67 leads to a significant increase in indel mutations, our analysis revealed an upstream homopolymeric nucleotide stretch of five adenines (spanning RT codons 48–50) that also resulted in increased indels. This observation was consistent among all HIV-1 variants, supporting its legitimacy. These data are congruent with the dramatically increased indels associated with HIV_TAM_ infection and the ability of HIV_TAMK65K_ and HIV_TAMK66K_ to rescue this defect being a component of the underlying mechanism by which silent mutations can alleviate fitness defects conferred by D67N/K70R.

Drug resistance mutations change the primary sequence of HIV-1, which can alter the length of homopolymeric stretches of nucleotides, as is the case for D67N in subtype B. Mononucleotide tracts are known to promote template switching, misalign the template-primer and result in single nucleotide deletions or misincorporations (57,61–62). HIV-1 RT template-switching may occur as the enzyme exhibits a tendency to pause at this homopolymeric stretch of eight nucleotides (19). Template-switching could lead to an increase in recombination (62), although this was not evaluated in our study. The propensity of HIV-1 RT to pause at homopolymeric regions in both DNA and RNA templates is mediated by the template sequence rather than either primer position, RT subtype origin, or RT concentration (19,61). Our findings support the observation that stretches of iterated nucleotides do not normally extend beyond six in the HIV-1 genome because they are likely deleterious to the virus.

The emergence of synonymous mutations in the RNA genome can have either deleterious or advantageous effects on viral fitness. The genomes of RNA viruses feature numerous functional properties beyond protein coding that are critical in their replication cycle. These regions show strong purifying selection against synonymous mutations suggesting their functional role in virus replication (63,64). Some of these functions, including alternative translation initiation, splicing reactions, genome packaging and replication rely on secondary or higher order RNA structures (65,66). Synonymous mutations that alter these *cis*-acting elements are often detrimental to virus replication (67). Other synonymous mutations have also been linked to advantages that lead to their selection in viral RNA genomes. For example, the synonymous mutation, A759G, becomes fixed in quasispecies of the ssRNA virus, puumala virus, conferring a transmission advantage to the virus in rodent populations (68). Similarly, the synonymous mutation A1869G, selected in cell culture, improves the genetic stability of the nonsynonymous 627K mutation, a known mammalian adaptation motif of the highly pathogenic clade 2.2 Eurasian lineage H5N1 avian influenza virus (69). However, the mechanisms by which these silent mutations confer an advantage at the molecular level remain to be elucidated.

Our study provides the first evidence that silent mutations in the HIV-1 subtype B RT selected during drug therapy *in vivo* can directly alleviate fitness defects in virus concurrently encoding the TAMs, D67N/K70R. The sharp increase in indel frequency due to TAMs is dramatically rescued with the introduction of a silent mutation at codon 65 or 66 of the HIV-1 subtype B RT template, supporting their role as novel compensatory mutations. To the best of our knowledge, this mechanism has not been previously reported in the literature, nor has the use of next-generation sequencing been employed to this effect. We postulate that the fitness defect associated with increased indel frequency may occur at other homopolymeric stretches of nucleotides in connection with RT pausing. This study presents compelling evidence for investigating the selection of other synonymous mutations that may act as compensatory mutations that contribute to increased viral fitness.

## SUPPLEMENTARY DATA

Supplementary Data are available at NAR Online.

SUPPLEMENTARY DATA
