# Supplementary material for: Silent mutations at codons 65 and 66 in reverse transcriptase alleviate indel formation and restore fitness in subtype B HIV-1 containing D67N and K70R drug resistance mutations
Source: Nucleic Acids Res. 2015 Mar 12;43(6):3256–71. doi: 10.1093/nar/gkv128 (PMC4381058; doi:10.1093/nar/gkv128)
Supplement: SUPPLEMENTARY DATA [file supp_43_6_3256__index.html]

Silent mutations at codons 65 and 66 in reverse transcriptase alleviate indel formation and restore fitness in subtype B HIV-1 containing D67N and K70R drug resistance mutations — Silent mutations at codons 65 and 66 in reverse transcriptase alleviate indel formation and restore fitness in subtype B HIV-1 containing D67N and K70R drug resistance mutations — SUPPLEMENTARY DATA 

# Silent mutations at codons 65 and 66 in reverse transcriptase alleviate indel formation and restore fitness in subtype B HIV-1 containing D67N and K70R drug resistance mutations

## SUPPLEMENTARY DATA

**Files in this Data Supplement:**

- SUPPLEMENTARY DATA
