# Supplementary material for: Silent mutations at codons 65 and 66 in reverse transcriptase alleviate indel formation and restore fitness in subtype B HIV-1 containing D67N and K70R drug resistance mutations
Source: Nucleic Acids Res. 2015 Mar 12;43(6):3256–71. doi: 10.1093/nar/gkv128 (PMC4381058; doi:10.1093/nar/gkv128)
Supplement: SUPPLEMENTARY DATA [file supp_gkv128_nar-03690-z-2014-File009.pdf]

SUPPLEMENTARY DATA

For the manuscript "Silent mutations in HIV-1 subtype B reverse transcriptase restore viral fitness and alleviate an increase in indels due to drug resistance mutations" by

Sushama Telwatte, Anna C. Hearps, Adam Johnson, Catherine F. Latham, Katie Moore, Paul Agius, Mary Tachedjian, Secondo Sonza, Nicolas Sluis-Cremer, P. Richard Harrigan, Gilda Tachedjian

SUPPLEMENTAL MATERIALS AND METHODS

Quantitative Western Blot Analysis

For analysis of viral proteins, clarified culture supernatants from transfections were subjected to ultracentrifugation at 53 000 x *g* for 1 h at 4°C through a 25% (w/v) sucrose cushion. The viral pellets were lysed with TNEN buffer (50 mM Tris pH 8.0, 50 mM NaCl, 10 mM EDTA and 0.5% IGEPAL) containing 1 µg/ml each of aprotinin, leupeptin and pepstatin and proteins resolved on a 10% sodium dodecyl sulfate-polyacrylamide (SDS-PAGE) gel (1). Proteins were transferred to Hybond C-extra nitrocellulose membrane (Amersham Biosciences, Piscataway, NJ) followed by detection using quantitative Western blot analysis that was performed using the Odyssey Infrared Imaging System as previously described (2). Pooled sera from HIV-infected individuals, kindly provided by Dale McPhee (National Serology Reference Laboratory, Australia), was diluted 1:10,000 to detect HIV-1 capsid. The monoclonal antibody 5B2, generously provided by Dag Helland (University of Bergen, Norway), that recognizes HIV-1 RT epitope 1 (codons 193 to 284) (3) was used to detect HIV-1 RT at a 1:100 dilution. Secondary antibodies used were IRDye 800CW conjugated affinity purified anti-human IgG (Rockland) and Alexa Fluor 680 Goat anti-mouse IgG (Invitrogen Life Technologies). Densitometry analysis was performed using the Odyssey Software v3.1 (Li-Cor, Lincoln, NE) as previously described (1).

qPCR-Based Product-Enhanced Reverse Transcriptase (qPERT) Assay

RT activity was quantified using the SYBR-Green I PERT (SG-PERT) assay for the ABI7300 protocol as described in (4) modified to use the Brilliant II SYBR Master Mix (Agilent Technologies). Briefly, 10 µl of diluted sample or recombinant avian myeloblastosis virus-RT (AMV-RT) (Promega, Madison, WI) was added to a qPCR master mix containing 1x Brilliant II SYBR Master Mix, 0.2 µM each of PERT A and PERT B primers, 80 ng of MS2 phage RNA (Roche, Basel, Switzerland), 0.1 µl of RNAsin (Promega) [diluted 1:10] and 1.8 µl of nuclease free water. The following cycling conditions were used on a Stratagene Mx3000 instrument (Agilent Technologies): 30 min RT reaction at 37°C, 10 min Brilliant II enzyme activation at 95°C followed by 40 cycles at 95°C for 30 sec, 60°C for 1 min and 72°C for 30 sec. Data were analyzed using MxPro software (Agilent Technologies).

SUPPLEMENTAL REFERENCES

1. Figueiredo, A., Moore, K.L., Mak, J., Sluis-Cremer, N., de Bethune, M.P. and Tachedjian, G. (2006) Potent nonnucleoside reverse transcriptase inhibitors target HIV-1 Gag-Pol. *PLoS Pathog.*, **2**, e119.
2. Telwatte, S., Moore, K., Johnson, A., Tyssen, D., Sterjovski, J., Aldunate, M., Gorry, P.R., Ramsland, P.A., Lewis, G.R., Paull, J.R. *et al.* (2011) Virucidal activity of the dendrimer microbicide SPL7013 against HIV-1. *Antiviral Res.*, **90**, 195-199.
3. Szilvay, A.M., Nornes, S., Haugan, I.R., Olsen, L., Prasad, V.R., Endresen, C., Goff, S.P. and Helland, D.E. (1992) Epitope mapping of HIV-1 reverse transcriptase with monoclonal antibodies that inhibit polymerase and RNase H activities. *J. Acquir. Immune. Defic. Syndr.*, **5**, 647-657.
4. Vermeire, J., Naessens, E., Vanderstraeten, H., Landi, A., Iannucci, V., Van Nuffel, A., Taghon, T., Pizzato, M. and Verhasselt, B. (2012) Quantification of reverse transcriptase activity by real-time PCR as a fast and accurate method for titration of HIV, lenti- and retroviral vectors. *PLoS One*, **7**, e50859.

SUPPLEMENTAL FIGURES AND LEGENDS

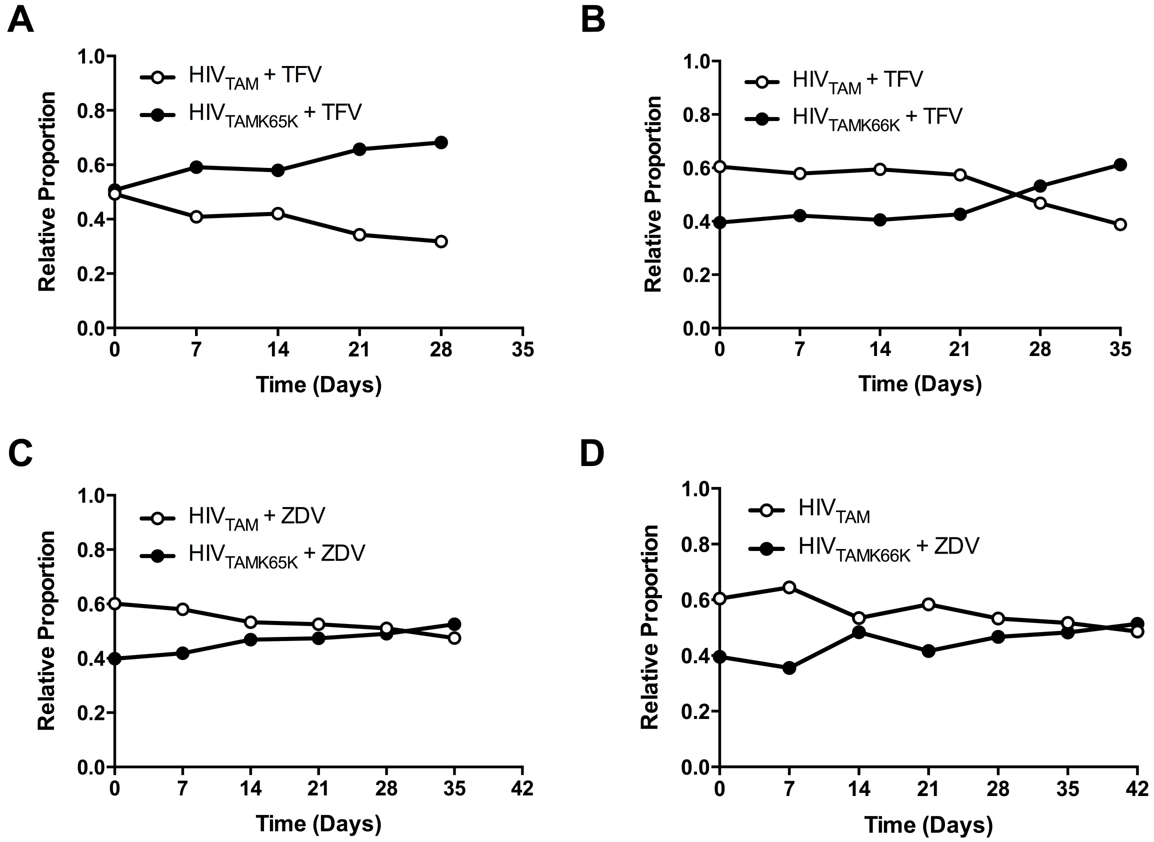

Figure S1. Growth competition dynamics between HIV_{TAM} and $HIV_{TAMK65K}$ or $HIV_{TAMK66K}$ in the presence of RT inhibitors performed in human peripheral blood mononuclear cells. Growth competition assays performed with HIV_{TAM} and HIV_{K65K} or HIV_{TAM} and $HIV_{TAMK66K}$ passaged in the presence of either (A, B) 5 µM tenofovir (TFV) or (C, D) 1 µM zidovudine (ZDV), respectively. Representative data are shown from n=2 independent assays for each condition.

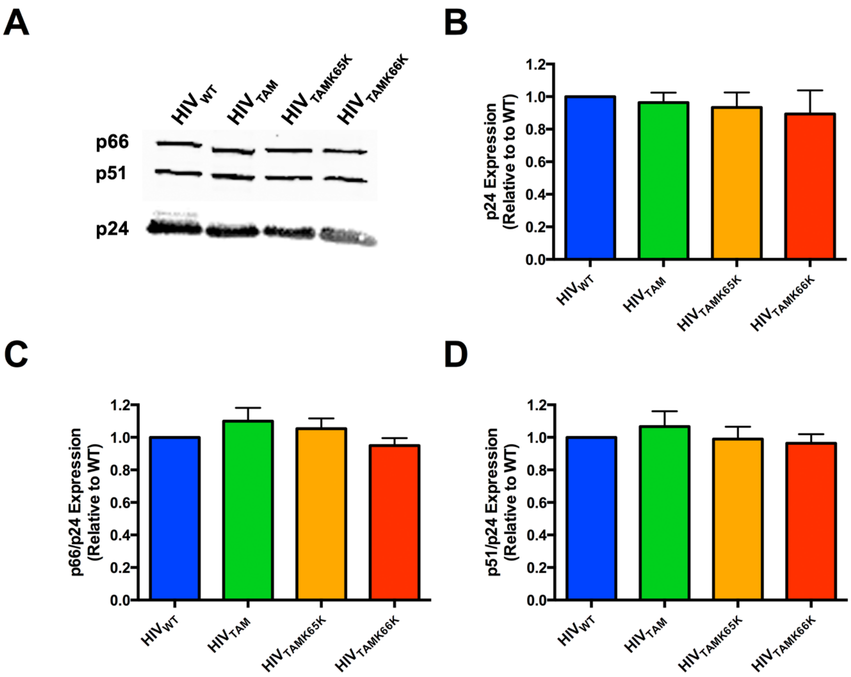

Figure S2. Relative expression of virion-associated reverse transcriptase and p24 capsid in HIV-1 generated by transfection. Cell-free supernatants were harvested 48 h post-transfection of 293T cells with $pHIV_{WT}$, $pHIV_{TAM}$, $pHIV_{TAMK65K}$ and $pHIV_{TAMK66K}$, virus was concentrated by ultracentrifugation through a sucrose cushion, lysed and subjected SDS-PAGE. **(A)** Western blot of RT subunits (p66 and p51) detected with the 5B2 primary antibody and p24 capsid detected using pooled sera from HIV positive individuals. Quantitative Western blot analysis of: **(B)** virion-associated p24 capsid relative to HIV_{WT}, **(C)** virion-associated p66 RT relative to HIV_{WT}, **(D)** and virion-associated p51 RT relative to HIV_{WT}. RT expression was normalized to virion-associated p24. Data represent mean ± standard error of the mean (SEM) from n=3 independent assays. No significant differences in relative viral protein expression were detected between HIV_{WT} and mutant viruses (*p*=0.86 for p24, *p*=0.39 for p66/p24 and *p*=0.84 for p51/p24) using the Kruskal-Wallis test with post Dunn's correction.

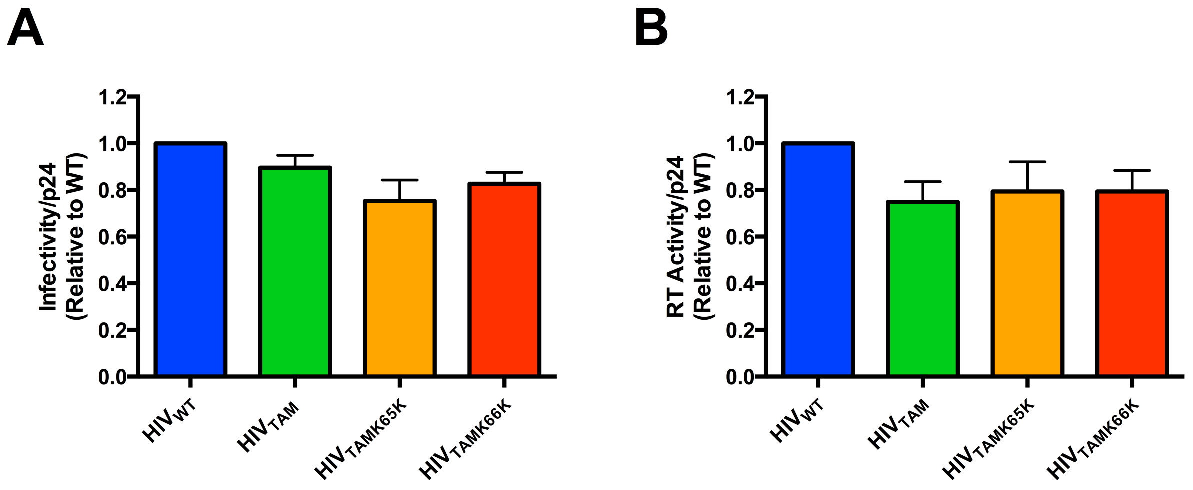

Figure S3. Infectivity and virion-associated RT activity of HIV-1 generated by transfection. Cell-free supernatants were harvested 48 h post-transfection of 293T cells with $pHIV_{WT}$, $pHIV_{TAM}$, $pHIV_{TAMK65K}$ and $pHIV_{TAMK66K}$. **(A)** Viral infectivity was determined in TZM-bl cells and normalized to p24, which was quantified by ELISA and expressed relative to HIV_{WT} (n=6 independent assays, mean ± SEM shown). **(B)** virion-associated RT activity was determined by product-enhanced reverse transcriptase (q-PERT) assay, normalized to p24 and expressed relative to HIV_{WT} (n=5 independent assays, mean ± SEM shown). No significant differences in relative RT activity (p=0.22) or infectivity (p=0.87) were observed between HIV_{WT} and mutant viruses using the Kruskal-Wallis test with post Dunn's correction.

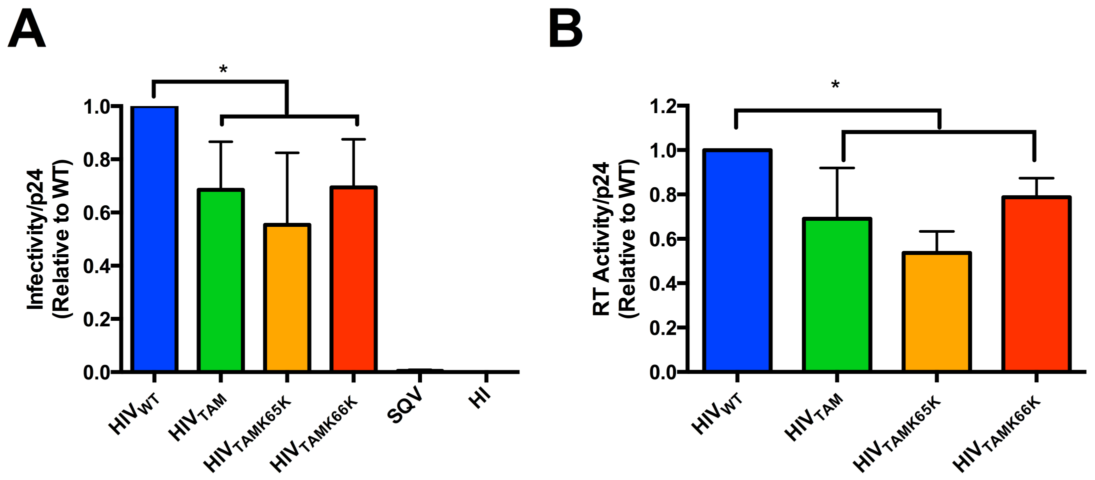

Figure S4. Production of infectious virus and virion-associated RT activity from single-cycle infection assays. Virus in cell-free supernatants was harvested 30 h post-infection of MT-2 cells. **(A)** The infectivity of HIV-1 from culture supernatants was determined in TZM-bl cells, normalized to p24 (quantified by ELISA) and expressed relative to HIV_{WT}. SQV denotes saquinavir-treated cells and HI denotes the heat-inactivated virus control. Bars indicate mean ± SEM and (*) denotes p=0.01. No significant differences were observed among the HIV-1 mutants (p>0.05). **(B)** Virion-associated RT activity was determined by q-PERT and normalized to p24. (*) denotes p=0.04. No significant differences were observed between the HIV-1 mutants (p>0.05). Kruskal-Wallis tests with post Dunn's correction were performed for each analysis.

SUPPLEMENTAL TABLES

Table S1. Overall Mutation Frequency During Single- and Multiple Round HIV-1 Replication

Template	Gross Frequency*	Net Frequency#
**$pHIV_{WT}$‡**	0.028	-
**$pHIV_{TAM}$‡**	0.017	-
**$pHIV_{TAMK65K}$‡**	0.016	-
**$pHIV_{TAMK66K}$‡**	0.023	-
**HIV_{WT}†**	0.087	0.059
**HIV_{TAM}†**	0.040	0.023
**$HIV_{TAMK65K}$†**	0.021	0.004
**$HIV_{TAMK66K}$†**	0.022	0.0
**HIV_{WT_L}¶**	0.075	0.047
**HIV_{TAM_L}¶**	0.118	0.101
**$HIV_{TAMK65K\_L}$¶**	0.054	0.036
**$HIV_{TAMK66K\_L}$¶**	0.064	0.041

* Sum of the frequency of all mutations (i.e. indels, MNV and SNVs) determined by paired-end Illumina next-generation amplicon sequencing divided by total length of the sequenced amplimer (108 bp).
# Subtraction of gross frequency for matching plasmid template control from the gross frequency of mutations generated in HIV-1 RT.
‡ Plasmid control templates with matching RT sequences compared to HIV-1 used in culture experiments.
† HIV-1 subjected to a single-cycle infection in MT-2 cells.
¶ HIV-1 subjected to long-term infection (19 days) in MT-2 cells.

Table S2. Frequency (%) of mutations in HIV-1 RT introduced during single-cycle infection in MT-2 cells

		HIV_{WT}				HIV_{TAM}				$HIV_{TAMK65K}$				$HIV_{TAMK66K}$			
**RT Codon**	**Nucleotide**†	**Insertions**¶	**Deletions**¶	**SNV***	**MNV**#	**Insertions**	**Deletions**	**SNV**	**MNV**	**Insertions**	**Deletions**	**SNV**	**MNV**	**Insertions**	**Deletions**	**SNV**	**MNV**
**64**	2741	0	0	0.034	0	0	0.00515	0.05	0	0	0	0.02	0	0	0	0.02	0
**65**	2742	0.02	0	0	0	1.91	0.11	0.00024	0	0	0	0	0	0.00729	0.01	0	0
	2743	0	0	0	0	0	0	0.0015	0	0	0	0	0	0	0	0	0
	2744	0	0	0.06	0.3	0	0	0	0	0	0	0	0.16	0	0	0	0
**66**	2745	0	0	0	0	0	0	0.00315	0	0.002	0	0	0	0	0	0.0036	0
	2746	0	0	0.00236	0	0	0	0.00778	0	0	0	0.002	0	0	0	0.00318	0
	2747	0	0	0.02	0.03	0	0	0.04	0	0	0	0	0	0	0	0.01	0
**67**	2748	0	0	2.37	0	0	0	0.03	0	0	0	0	0	0	0	0	0
	2749	0	0	0.002	0	0	0	0	0	0	0	0.0052	0	0	0	0	0
	2750	0	0	0.014	0	0	0	0.01663	0	0	0	0	0	0	0	0.007	0
**68**	2751	0	0	0	0	0	0	0	0	0	0	0	0	0	0	0.00149	0
	2752	0	0	0.0016	0	0	0	0.012	0	0	0	0.01	0	0	0	0.01	0
	2753	0	0	0	0	0	0	0.0056	0	0	0	0.00329	0	0	0	0.0056	0
**69**	2754	0	0	0	0	0	0	0	0	0	0	0.00158	0	0	0	0	0
	2755	0	0	0.01604	0	0	0	0.00555	0	0	0	0.01	0	0	0	0.011	0
	2756	0	0	0.0084	0	0	0	0	0	0	0	0.00746	0	0	0	0	0
**70**	2757	0	0	0	0	0	0	0.0089	0	0	0	0.00622	0	0	0	0.004	0
	2758	0	0	3.2	0	0	0	0.04	0	0	0	0.02	0	0	0	0.01	0
	2759	0	0	0.005	0	0	0	0.00302	0	0	0	0	0	0	0	0.0025	0

† Nucleotides numbered from 2741 – 2759 relative to HXB2 corresponding to RT codons 64 – 70.
¶ Frequency of insertions and deletions. Data shown is the sum of all insertions (i.e. single, double, triple and quadruple) or deletions at a particular nucleotide.
* Frequency of single nucleotide variants (SNV). Data shown is the sum of all changes at a particular nucleotide.
# Frequency of multiple nucleotide variants (MNV). Data shown is the sum of all changes at a particular nucleotide.

Table S3. Frequency (%) of mutations in HIV-1 RT introduced during multiple-cycle infection in MT-2 cells

		HIV_{WT_L}				HIV_{TAM_L}				$HIV_{TAMK65K\_L}$				$HIV_{TAMK66K\_L}$			
**RT Codon**	**Nucleotide**[†]	**Insertions**[¶]	**Deletions**[¶]	**SNV***	**MNV**[#]	**Insertions**	**Deletions**	**SNV**	**MNV**	**Insertions**	**Deletions**	**SNV**	**MNV**	**Insertions**	**Deletions**	**SNV**	**MNV**
**64**	2741	0	0.00805	0.1636	0	0	0.02	0.24	0.0056	0	0	0.18	0	0	0	0.2	0.00738
**65**	2742	0.09	0.02	0.01	0	**6.42**	0.26	0.00024	0	0.00985	0	0.00551	0	0.03	0.03	0	0
	2743	0	0	0.01	0	0	0	0.00204	0	0	0	0.0015	0	0	0	0.01	0
	2744	0	0	0.06	0.02	0	0	0	0	0	0	0.39548	0.03679	0	0	0.02	0
**66**	2745	0	0	0.004	0	0	0	0.004	0	0.00968	0.012	0	0	0	0	0.00351	0
	2746	0	0	0.00236	0	0	0	0.0046	0	0	0	0.022	0	0	0	0.22	0
	2747	0	0	0.05	0.00673	0	0	0.03	0	0	0	0.04	0	0	0	0.02	0
**67**	2748	0	0	0.39	0	0	0	0	0.03	0	0	0	0	0	0	0.01566	0
	2749	0	0	0.011	0	0	0	0	0	0	0	0.01195	0	0	0	0.002	0
	2750	0	0	0.014	0	0	0.00846	0.085	0	0	0	0.06	0	0	0	0.0047	0
**68**	2751	0	0	0	0	0	0	0.00033	0	0	0	0.005	0	0	0	0.00965	0
	2752	0	0	0.0016	0	0	0	0.012	0	0	0	0.06638	0	0	0	0.10804	0
	2753	0	0	0.04004	0	0	0	0.03311	0	0	0	0.03627	0	0	0	0.016	0
**69**	2754	0	0	0.00401	0	0	0	0.00919	0	0	0	0.0173	0	0	0	0.01576	0
	2755	0	0	0.021396	0	0	0	0.045	0	0	0	0.03	0	0	0	0.0439	0
	2756	0	0	0.03625	0	0	0	0.07574	0	0	0	0.03775	0	0	0	0.04	0
**70**	2757	0	0.00067	0.0079	0	0	0	0.0081	0	0	0	0.00214	0	0	0	0.00399	0
	2758	0	0	0.29	0	0	0	0.04	0.05171	0	0	0.17	0.02563	0	0	0.09524	0.04837
	2759	0	0	0.009	0	0	0	0.014	0	0	0	0.01702	0	0	0	0.0037	0

[†] Nucleotides numbered from 2741 – 2759 relative to HXB2 corresponding to RT codons 64 – 70.

[¶] Frequency of insertions and deletions. Data shown is the sum of all insertions (i.e. single, double, triple and quadruple) or deletions at a particular nucleotide.

* Frequency of single nucleotide variants (SNV). Data shown is the sum of all changes at a particular nucleotide.

[#] Frequency of multiple nucleotide variants (MNV). Data shown is the sum of all changes at a particular nucleotide
